# Two HIV-1 Variants Resistant to Small Molecule CCR5 Inhibitors Differ in How They Use CCR5 for Entry

**DOI:** 10.1371/journal.ppat.1000548

**Published:** 2009-08-14

**Authors:** Reem Berro, Rogier W. Sanders, Min Lu, Per J. Klasse, John P. Moore

**Affiliations:** 1 Department of Microbiology and Immunology, Weill Medical College of Cornell University, New York, New York, United States of America; 2 Department of Biochemistry, Weill Medical College of Cornell University, New York, New York, United States of America; Harvard Medical School, United States of America

## Abstract

HIV-1 variants resistant to small molecule CCR5 inhibitors recognize the inhibitor-CCR5 complex, while also interacting with free CCR5. The most common genetic route to resistance involves sequence changes in the gp120 V3 region, a pathway followed when the primary isolate CC1/85 was cultured with the AD101 inhibitor *in vitro*, creating the CC101.19 resistant variant. However, the D1/86.16 escape mutant contains no V3 changes but has three substitutions in the gp41 fusion peptide. By using CCR5 point-mutants and gp120-targeting agents, we have investigated how infectious clonal viruses derived from the parental and both resistant isolates interact with CCR5. We conclude that the V3 sequence changes in CC101.19 cl.7 create a virus with an increased dependency on interactions with the CCR5 N-terminus. Elements of the CCR5 binding site associated with the V3 region and the CD4-induced (CD4i) epitope cluster in the gp120 bridging sheet are more exposed on the native Env complex of CC101.19 cl.7, which is sensitive to neutralization via these epitopes. However, D1/86.16 cl.23 does not have an increased dependency on the CCR5 N-terminus, and its CCR5 binding site has not become more exposed. How this virus interacts with the inhibitor-CCR5 complex remains to be understood.

## Author Summary

Human immunodeficiency virus type 1 (HIV-1) is the causative agent of AIDS. HIV-1 entry into target cells is triggered by the interaction of the viral envelope glycoproteins with a cell-surface receptor (CD4) and a co-receptor (CCR5), and culminates in fusion of the viral and cell membranes. Small molecule inhibitors that bind to CCR5 are a new class of drug for treating HIV-1-infected people. However, HIV-1 can evolve ways to become resistant to these compounds, by acquiring mutations that alter how its envelope glycoproteins (gp120-gp41) interact with CCR5. In this study, we investigated how two resistant viruses gained the ability to use the inhibitor-bound form of CCR5 through two different mechanisms. In the first virus, four amino acid substitutions in the V3 region of gp120 created an increased dependency on interactions with the CCR5 N-terminus. These changes altered the configuration of gp120, increasing the exposure of antibody epitopes in the V3 region and the CD4i epitope cluster associated with the CCR5 binding site. In contrast, the second virus, which became resistant via three sequence changes in the gp41 subunit, did not become more dependent on the CCR5 N-terminus and remained resistant to neutralization by antibodies against elements of the CCR5 binding site.

## Introduction

Small molecule drugs or drug candidates bind to the cell surface CCR5 protein and prevent human immunodeficiency virus type 1 (HIV-1) from using it as a coreceptor for entry into CD4-positive target cells [1],[2]. These compounds, which include the licensed drug maraviroc (MVC) and the clinical candidate vicriviroc (VVC, also known as SCH-D), bind within the transmembrane helices of CCR5 and stabilize the protein in a conformation that cannot be recognized efficiently by the HIV-1 gp120 surface glycoprotein [3]–[7]. The interaction between gp120 and CCR5 is considered to involve two structural elements. The CCR5 N-terminus (NT) interacts with a site on gp120 that involves the 4-stranded bridging sheet region and the base of V3, which assembles upon CD4 binding, while the second extracellular loop (ECL-2) of CCR5 interacts with a second region of V3 located near its tip [8]–[12].

Viruses resistant to the small molecule CCR5 inhibitors can be generated *in vitro* and *in vivo* [13]–. The dominant route to resistance involves the acquisition of sequence changes that render gp120 capable of recognizing the inhibitor-CCR5 complex, without losing its ability to also interact with the free coreceptor [16],[18]. Hence the escape mutants become inhibitor-tolerant, but not inhibitor-dependent. The most common genetic route to resistance is the acquisition of multiple sequence changes in V3 [13], [16], [19]–[21]. This pathway was followed when the primary R5 isolate CC1/85 was cultured with the AD101 inhibitor *in vitro*, creating the CC101.19 resistant variant. However, we have described a V3-independent route to the same phenotype that was taken by the same input virus under the selection pressure of a similar compound, VVC, to yield the D1/86.16 escape mutant [14],[22]. We have recently shown that this alternative pathway involves three sequence changes in the fusion peptide (FP) region of the gp41 transmembrane glycoprotein. These changes exert broadly similar effects to the more conventional V3 changes, in that the resistant virus was able to use the inhibitor-CCR5 complex for entry [22].

By using CCR5 point-mutants and gp120-targeting agents, we now seek to learn more about how the parental and both resistant viruses interact with the coreceptor. A small molecule that interacts with gp120 at the V3 region, IC9564, had differential activities against the various viruses, as did monoclonal antibodies (MAbs) and polyclonal Abs directed against the V3 region and MAbs to the CD4-induced epitopes associated with CCR5 binding. We conclude that the V3 sequence changes in CC101.19 create a variant that is more dependent than its parent on interactions with the CCR5 NT. Elements of the CCR5 binding site associated with the V3 region and the CD4i epitope cluster in the bridging sheet have become more exposed on the native Env complex of this virus, and hence accessible to neutralizing antibodies (NAbs). However, the D1/86.16 variant with changes in the gp41 FP has followed a different pathway to resistance that does not involve an increased dependency on the CCR5 NT, and in which the CCR5 binding site has not become more exposed. How this virus interacts with the inhibitor-CCR5 complex therefore remains to be determined.

## Results

### Differential usage of CCR5 mutants by parental and escape mutant viruses

**Figure 1:**
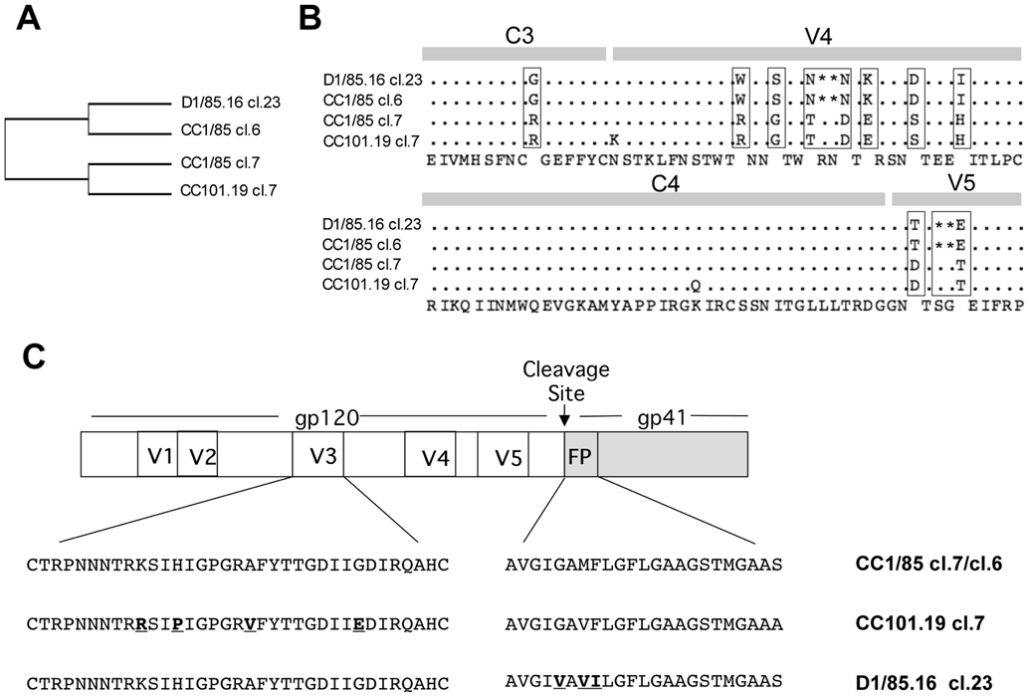
Sequence analysis of parental and resistant viruses. (A) Tree view of a multiple Env amino acid sequence alignment of the CC1/85-derived clones (cl.6, cl.7) and the two CCR5 inhibitor-resistant clones, CC101.19 cl.7 and D1/85.16 cl.23. (B) Alignment of gp120 sequences from residues 370–470, consisting of a segment of C3, V4, C4 and V5. The consensus amino acid sequence is given on the bottom line. Dots represent identical residues for all four clones, with gaps indicated by asterisks. Residues that are different between the two pairs (D1/85.16 cl.23 and CC1/85 cl.6 on the one hand, CC101.19 cl.7 and CC1/85 cl.7 on the other) are boxed. (C) Schematic representation of HIV-1 Env clones. The V3 and FP sequences of representative clones of CC1/85, CC101.19 and D1/85.16 are depicted. CC101.19 cl.7 contains four substitutions in the V3 region of gp120 (K305R, H308P, A316V and G321E), while D1/85.16 cl.23 contains three substitutions in the gp41 FP (G516V, M518V and F519I) [20],[22]. These sequence differences are highlighted in bold and underlined; they are necessary and sufficient to confer resistance, although there are other changes elsewhere in Env. Amino acid numbering is based on HxB2 Env.

Isolates CC101.19 and D1/85.16 are resistant variants derived from the primary R5 isolate CC1/85 after selection with the small molecule CCR5 inhibitors AD101 and VVC, respectively [14],[20]. As the emphasis of the present study was to gain a better understanding of how resistant variants interact with CCR5, we used infectious, Env-chimeric clonal viruses CC101.19 cl.7 and D1/85.16 cl.23, derived from the above resistant isolates, and compared their properties with inhibitor-sensitive clones of the parental isolate, CC1/85. A multiple sequence alignment based on the Env amino-acid sequences of seven parental clones derived from the CC1/85 isolate shows that CC1/85 cl.7 and CC1/85 cl.6 were the most similar to CC101.19 cl.7 and D1/85.16 cl.23, respectively (data not shown). For simplicity, we have summarized these results in a tree based on the percent similarity between the four clones (Fig. 1A,B). The majority of the amino-acid differences between the two pairs of viral clones are in the V4 and V5 regions of gp120. Taking into account also the replication properties of the various parental clones, we chose to use CC1/85 cl.7 for comparisons with CC101.19 cl.7, and CC1/85 cl.6 as a comparator for D1/85.16 cl.23. Clones CC101.19 cl.7 and D1/85.16 cl.23 contain amino acid changes that have been shown to be necessary and sufficient to confer resistance to small molecule CCR5 inhibitors [20],[22]. Thus, CC101.19 cl.7 has four substitutions in the V3 region of gp120 (K305R, H308P, A316V and G321E), while D1/85.16 cl.23 contains three changes in the gp41 FP (G516V, M518V and F519I) (Fig. 1C). The phenotypic properties of these four clones that were derived from the studies outlined below are summarized in Table S1.

**Figure 2:**
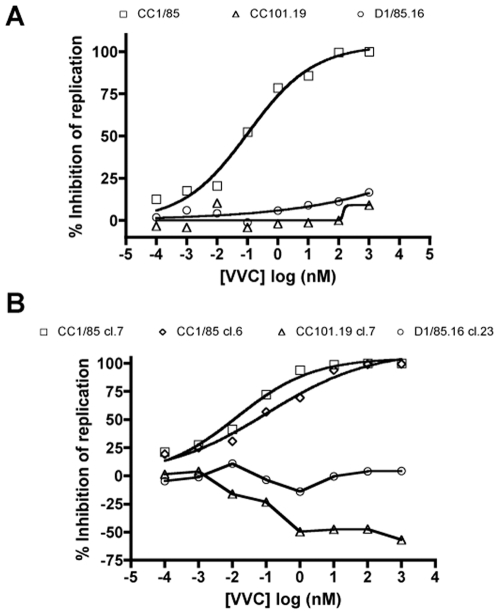
VVC sensitivity of parental and resistant isolates and clones. (A) The parental isolate CC1/85 (squares) and the resistant isolates CC101.19 (triangles) and D1/85.16 (circles) were tested for VVC sensitivity in an assay of HIV-1 replication in PBMCs. (B) Clones derived from the parental isolate, CC1/85 cl.7 (squares) and CC1/85 cl.6 (diamonds), or from the resistant isolates, CC101.19 cl.7 (triangles) and D1/85.16 cl.23 (circles), were used to infect PBMCs in the presence of VVC. The data in both panels A and B represent the extent of inhibition of replication (p24 antigen production) relative to that in the absence of VVC (100% replication, 0% inhibition). The data points in panels A and B are derived from a single, representative experiment.

The four clones used in this study recapitulate the phenotypes of the corresponding isolates in respect of VVC sensitivity. Thus, in an assay using PBMCs, the parental isolate, CC1/85, was completely inhibited by VVC concentrations ≥100 nM, whereas replication of the two resistant isolates was not affected by the presence of VVC (Fig. 2A). Similarly, the parental clones CC1/85 cl.7 and CC1/86 cl.6 were each completely inhibited by VVC concentrations ≥10 nM (10-fold lower then that needed for complete inhibition of the isolate). In contrast, replication of the resistant clones in PBMCs was either modestly enhanced (for CC101.19 cl.7) or unaffected (for D1/85.16 cl.23) by VVC (Fig. 2B). Env-pseudotyped viruses derived from the above clones behaved similarly to the infectious, chimeric clonal viruses in U87-CD4-CCR5 assays (data not shown). Similar data were obtained using other small molecule CCR5 inhibitors such as AD101, maraviroc and aplaviroc (data not shown).

**Figure 3:**
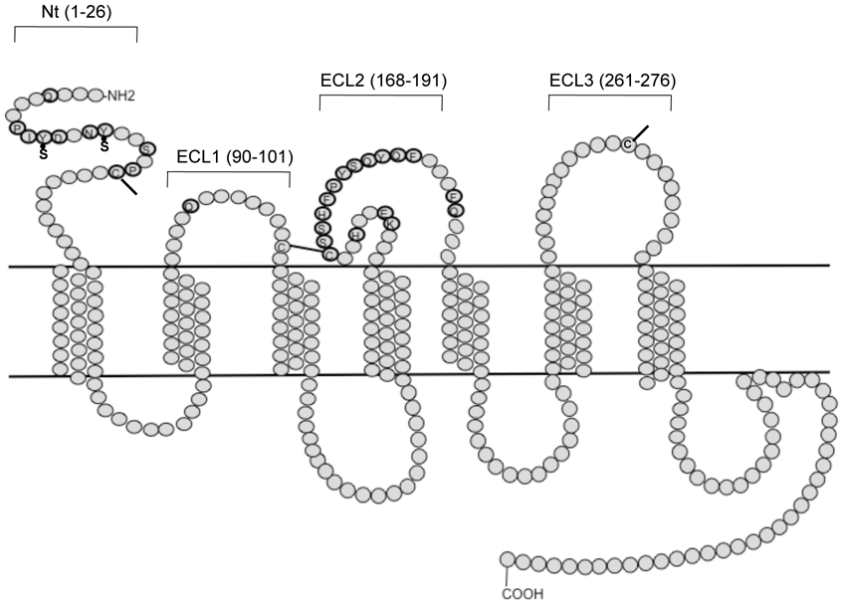
Schematic representation of CCR5 showing positions of amino acid changes in mutant panel. The locations of the NT and the extracellular loops are indicated. Residues altered in the various mutants are highlighted and labelled, and the positions of the critical sulfated tyrosine moieties in the NT are also shown. Bars at the Cys residues represent potential disulfide bonds between C101 and C178 and between C20 and C269, which are important for maintaining CCR5 in the correct conformation.

To determine whether the resistant clones differ from each other, and from the corresponding parental clone, in how they interact with CCR5, we first used a panel of point-mutated coreceptors (Fig. 3, Table 1). The composition of the test panel was biased towards mutants of the NT and ECL2, since these CCR5 domains have the greatest influence on HIV-1 entry [10], [23]–[25].

**Table 1:** Effect of CCR5 mutations on entry of parental and resistant Env-pseudotyped viruses*.

		CC1/85 cl.7	CC101.19 cl.7	CC1/85 cl.6	D1/85.16 cl.23
	**CCR5-WT**	100	100	100	100
***NT***	**Δ18**	**0**	**0**	**0**	**0**
	**Q4A**	84±11	90±9	72±4	75±15
	**P8A**	67±4	76±10	57±2	66±16
	**I9T**	89±16	86±17	94±5	81±2
	**Y10A**	80±18	**20±6**	60±4	60±13
	**D11A**	**38±12**	**18±7**	**50±3**	**41±5**
	**N13A**	71±6	73±11	74±5	70±21
	**Y14A**	**38±2**	**0**	62±1	69±6
	**Y14F**	55±11	**1±0**	61±4	73±21
	**Y14Q**	68±9	**20±11**	90±6	99±26
	**Y10A/Y14A**	**9±6**	**1±0**	**19±1**	**35±1**
	**S17A**	76±8	65±8	84±4	89±8
	**P19A**	70±3	69±4	64±0	58±8
	**C20A**	56±8	**26±9**	70±2	74±14
***TM2/ECL1***	**Q93A**	76±3	82±15	66±4	66±8
***ECL2***	**K171A**	116±4	104±3	92±2	105±9
	**E172A**	88±5	69±5	75±2	86±2
	**H175A**	86±12	94±12	83±2	81±18
	**C178A**	**14±4**	**28±8**	**20±1**	**29±6**
	**S179A**	93±8	89±6	90±11	91±4
	**S180A**	100±1	93±2	92±3	95±7
	**H181A**	80±8	54±5	68±5	58±8
	**F182A**	51±20	**27±6**	**46±5**	**28±16**
	**P183A**	58±15	**37±1**	59±8	**31±8**
	**YSQ184-186AAA**	59±11	62±5	52±1	55±1
	**Q186A**	97±10	98±27	115±6	84±13
	**YQF187-189AAA**	95±20	95±16	89±12	83±2
	**Y187A**	57±8	61±6	56±5	**37±5**
	**F189A**	**43±2**	**46±3**	**40±5**	**30±1**
	**F193A**	65±13	59±11	68±6	**49±20**
	**Q194A**	106±7	103±16	100±7	99±6

*: The values recorded are the relative entry efficiencies of the CC1/85 cl.7, CC101.19 cl.7, CC1/85 cl.6 and D1/85.16 cl.23 Env-pseudotyped viruses into U87-CD4 cells transiently expressing (for 48 h) the CCR5 proteins indicated. Luciferase expression was measured 72 h after infection, and is normalized relative to the entry of each virus via WT CCR5 (defined as 100%). Each value is the mean±SEM of at least three independent determinations. A 50% decrease in entry efficiency was considered to be meaningful in this assay, so all values ≤50% are bolded. It is possible that lesser decreases (e.g., 30–50%) might also be biologically relevant.

The various CCR5 mutants were transiently expressed in U87-CD4 cells for 48 h before incubation for an additional 72 h with luciferase-expressing, Env-pseudotyped clonal viruses derived from the parental and resistant isolates. The CCR5 mutants were all expressed at comparable levels on the cell surface as determined by FACS (data not shown). The relative level of entry via each mutant, compared to wild-type CCR5, was calculated for each test virus, to identify coreceptor variants that were used with different efficiency under the conditions of this single-cycle assay (Table 1). As expected, none of the Env-pseudotyped viruses could use the Δ18 mutant that lacked the first 18 residues of the CCR5 NT [26],[27]. The tyrosine residues at NT positions 10 and 14 are sulfated, a modification known to be important for HIV-1 entry [28],[29]. Accordingly, none of the viruses utilised the Y10A/Y14A double mutant efficiently, although D1/85.16 cl.23 was able to use it for low-level entry (Table 1). Three other mutations adversely affected entry of all four viruses to a meaningful extent (<50% entry compared to wild-type): D11A in the NT, C178A and F189A in ECL2 (Table 1). This outcome is consistent with previous studies on the same mutants with different test viruses, and arises because these residues (particularly D11 and C178) are important for maintaining the appropriate CCR5 conformation [25],[30]. The entry of various viruses via certain other mutants was reduced to a lesser extent (25–50%). Such reductions may be biologically relevant but can be difficult to distinguish from background variation with confidence.

Several mutations differentially affected entry of the four Env-pseudotyped viruses. Thus, CC101.19 cl.7 was particularly affected by NT mutations Y10A, Y14A, Y14F, Y14Q and C20A (Table 1); depending on the mutation, the entry of this virus was reduced to ≤26% of the extent conferred by wild-type CCR5. The identity of the substituted residue at position 14 was an additional variable; more specifically, CC101.19 cl.7 could use the Y14Q mutant with low efficiency (∼20%), but not Y14A or Y14F (<1% entry). In contrast, D1/85.16 cl.23 could enter via all three of the residue-14 mutants at >70% of the level mediated by wild-type CCR5; indeed, this escape mutant and its parent, CC1/85 cl.6, were little affected by the identity of the residue at position 14 (Table 1). These observations, taken together, suggest that the tyrosine residues at positions 10 and 14 were both required for efficient entry of CC101.19 cl.7, whereas the presence of either sulfated-tyrosine was sufficient to mediate entry of the other three viruses to at least some extent. Conversely, the ECL2 mutations F182A and P183A impaired entry of both resistant viruses a little more than they did the two parental clones, while the Y187A and F193A changes selectively, although modestly, affected entry of D1/85.16 cl.23 (Table 1). The triple Ala mutants with changes at residues 184–186 and 187–189 were, however, used by all four viruses (Table 1).

Overall, the pattern of entry via the various CCR5 mutants suggests that the two resistant viruses differ markedly in how they interact with the coreceptor. Thus, CC101.19 cl.7 is particularly reliant on the sulfated tyrosine residues at positions 10 and 14 in the NT, but this is not the case for D1/85.16 cl.23. The latter virus is somewhat more affected by some substitutions within ECL2, but not dramatically so. Their differential sensitivity to CCR5 mutations suggests that the two escape mutants differ in how they interact with the coreceptor. A corollary of the increased dependence of CC101.19 cl.7 on the CCR5 NT might be that the region near the tip of V3 is now less involved in gp120-CCR5 binding, compared to both of the parental clones and D1/85.16 cl.23. If so, the 4 amino acid changes in the V3 region of CC101.19 cl.7 might be acting to change the orientation of V3 with respect to the rest of gp120, disrupting its ability to interact with ECL2 while increasing the accessibility of the bridging sheet to the NT. This argument would not apply to D1/85.16 cl.23, which has followed a different route to resistance that is less apparent from the studies using the CCR5 mutants. To gain information on what changes in Env conformation took place as resistance developed, we measured the responses of the two resistant viruses to compounds that interact with different regions of gp120.

### Sensitivity to inhibitors of gp120-CD4 binding does not correlate with VVC resistance

**Figure 4:**
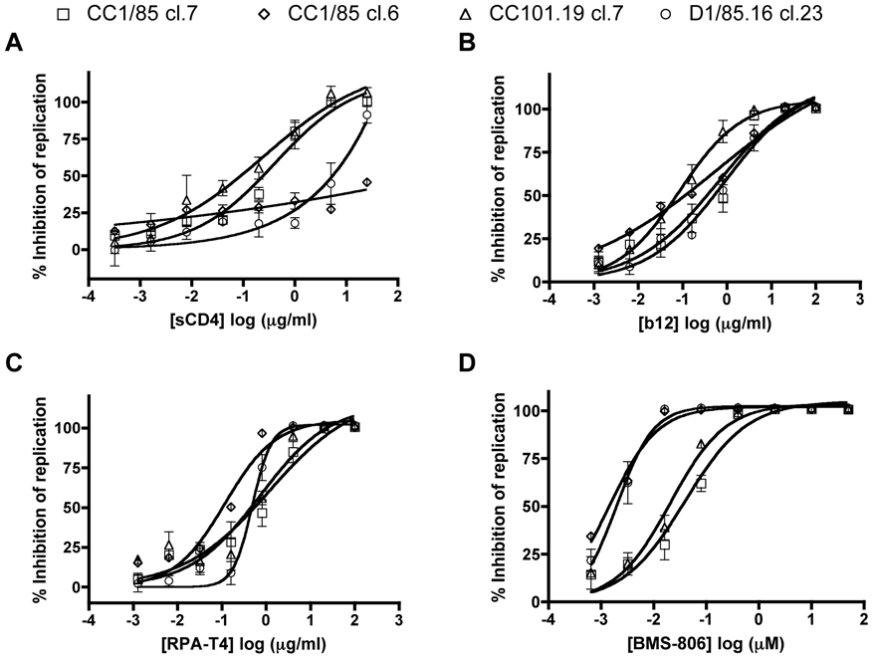
Sensitivity of parental and VVC-resistant clones to inhibitors of the gp120-CD4 interaction. Chimeric molecular clones CC1/85 cl.7 (squares), CC1/85 cl.6 (diamonds), CC101.19 cl.7 (triangles) and D1/85.16 cl.23 (circles) were used to infect PBMCs in the presence of the indicated concentrations of (A) sCD4, (B) MAb b12, (C) MAb RPA-T4 or (D) BMS-806. Of these compounds, sCD4, b12 and BMS-806 bind to gp120, RPA-T4 to CD4, but each of them inhibits gp120-CD4 binding. The data represent the extent of inhibition of replication (p24 antigen production) relative to that in the absence of inhibitor (100% replication, 0% inhibition). The data points in all panels are mean values±SEM from 3 independent experiments.

We first used various inhibitors of the gp120-CD4 interaction to assess whether there are differences in the CD4-binding events of the VVC-sensitive and -resistant clones that could influence the subsequent conformational changes in gp120 involved in creation of the CCR5 binding site. When the four clones were incubated with a range of sCD4 concentrations before infection of PBMCs, CC1/85 cl.7 and CC101.19 cl.7 were both highly sensitive, with IC_50_ values ∼0.1 µg/ml (Fig. 4A, Table 2). In contrast, D1/85.16 cl.23 was ∼100-fold less sensitive to sCD4 and CC1/85 cl.6 was almost completely resistant (Fig. 4A, Table 2). Of note is that CC1/85 cl.7 and CC101.19 cl.7 are unusually sensitive to sCD4, compared to the corresponding isolates (IC_50_ values ∼5 µg/ml) and to typical primary isolates, which typically have IC_50_ values >10 µg/ml [31]–[34]. The same data pattern was observed with CD4-IgG2 (PRO542); again CC1/85 cl.7 and CC101.19 cl.7 were much more sensitive than D1/85.16 cl.23 and all three of the isolates (Table 2). Hence the sCD4 and CCR5 inhibitor sensitivity profiles of these four clones are not correlated; one parental and one VVC-resistant clone are sCD4-sensitive, the other two are sCD4-resistant. In contrast to what was observed using sCD4 and CD4-IgG2, the four clones (and the corresponding isolates) did not differ markedly in their sensitivities to MAb b12 against the CD4-binding site on gp120 or to the anti-CD4 MAb RPA-T4 that inhibits gp120-CD4 binding (Fig. 4B,C and data not shown).

**Table 2:** IC_50_ values for inhibition of parental and resistant clones and isolates by gp120-targeting compounds.

	*Clones*				*Isolates*		
	CC1/85 cl.7	CC101.19 cl.7	CC1/85 cl.6	D1/85.16 cl.23	CC1/85	CC101.19	D1/85.16
sCD4*	0.10±0.035	0.10±0.027	**>25**	**10±0.058**	>10	>10	>10
CD4-IgG2 (PRO542)*	0.12±0.045	0.1±0.017	ND	**8±0.95**	4.7±1.6	6.2±1. 7	2.6±0.90
BMS-806†	40±4.2	21±1.3	**1.5±0.40**	**2.0±0.12**	20±2.0	30±6.1	**3.0±1.7**
IC9564†	7.2±2.6	**0.62±0.10**	**150±10**	**690±86**	300±12	170±4.3	**1700±44**

*: IC_50_ values are expressed in µg/ml.

**†:** IC_50_ values are expressed in nM.

ND = Not done.

All IC_50_ are mean of 3 independent experiments±SEM.

Values in bold indicate that IC_50_ is significantly different (at least 5-fold) than that of CC1/85 cl.7 (for the clones) and CC1/85 (for the isolates).

**Figure 5:**
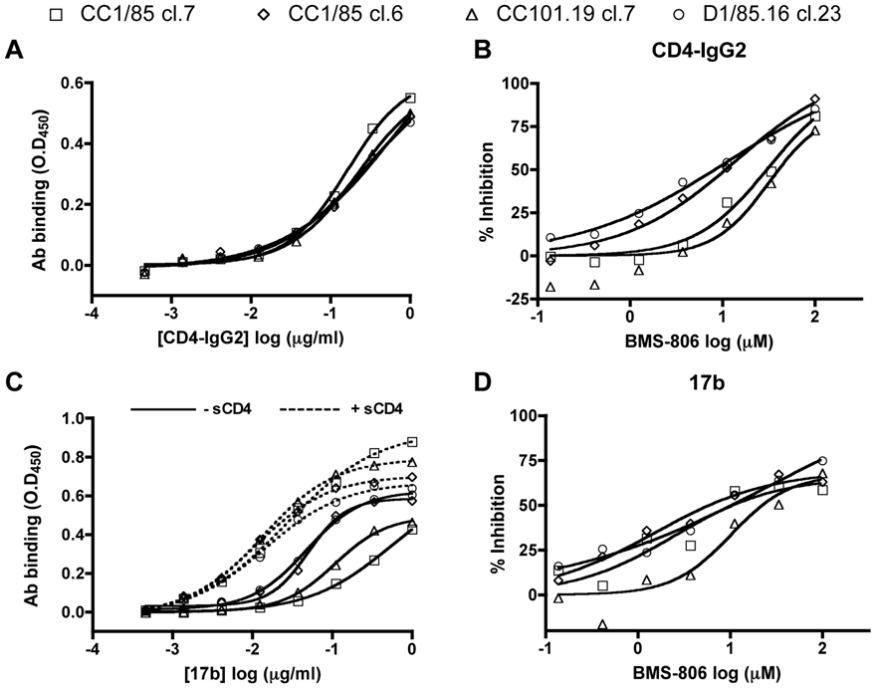
Binding of CD4-IgG2 and MAb 17b to monomeric gp120. Equal amounts of gp120 from viral lysates were captured by D7324 onto an ELISA plate and incubated with (A) CD4-IgG2; (B) CD4-IgG2 (0.4 µg/ml) plus increasing concentrations of the competitor, BMS-806; (C) MAb 17b in the absence or presence of sCD4 (300 ng/ml); (D) MAb 17b (0.4 µg/ml) plus increasing concentrations of the competitor, BMS-806. (A, C) The OD_450_ values shown were corrected for background binding of CD4-IgG2 and 17b, respectively, as measured in the absence of gp120. (B, D) The data shown represent the percentage inhibition of binding of CD4-IgG2 and 17b, respectively, in the presence of the indicated concentrations of BMS-806, with 0% inhibition (100% binding) occurring when BMS-806 was absent. The data points in each panel were derived from a single representative experiment.

The binding of MAbs and other ligands to gp120 monomers is usually not predictive of how the same agents interact with the native Env trimer and neutralize the corresponding virus [35]–[37]. However, because of the unusual characteristics of the CCR5 inhibitor resistant viruses, we considered it worth assessing whether the differential inhibition patterns described above might be manifested at the level of the gp120 monomer. In a gp120-capture ELISA, CD4-IgG2 bound with equivalent affinity to gp120 proteins derived from all four parental clones and resistant clones (Fig. 5A). Hence the differential sensitivity of the corresponding viruses to CD4-based inhibitors (Table 2) is not manifested at the level of the gp120 monomer, consistent with previous findings [35]–[37].

The small molecule HIV-1 gp120 ligand, BMS-806, was initially classified as an inhibitor of gp120-CD4 binding [38]. However, it also inhibits subsequent conformational changes in the gp120-gp41 complex [39]. It is not a direct competitor with gp120 for CD4 binding but instead reduces the affinity of CD4 for gp120 allosterically, without inducing the CD4i epitope [40]. The BMS-806 infectivity-inhibition pattern for the four clones was the converse of that seen with sCD4 (Fig. 4D, Table 2). Thus, D1/85.16 cl.23 and CC1/85 cl.6 were markedly more sensitive to BMS-806 than the other two clones (IC_50_ values ∼20-fold lower). D1/85.16 was also the most sensitive of the three isolates to BMS-806, by ∼7 to 10-fold (Table 2). We then tested whether the differential sensitivities of the viral clones to BMS-806 were also reflected at the gp120 monomer level. In an ELISA, BMS-806 inhibited the binding of CD4-IgG2 to gp120s from D1/85.16 cl.23 and CC1/85 cl.6 more efficiently than to gp120s from CC1/85 cl.7 and CC101.19 cl.7 (Fig. 5B). Hence the increased BMS-806 sensitivity of clones D1/85.16 cl.23 and CC1/85 cl.6 probably arises at the gp120 monomer level.

Given the similarities at the amino acid sequence level between CC1/85 cl.7 and CC101.19 cl.7 (Fig. 1A,B), it appears likely that CC101.19 cl.7 evolved from a sCD4-sensitive, minor variant present in the uncloned isolate that is related to CC1/85 cl.7. Conversely, D1/85.16 cl.23 presumably evolved from one of the more prevalent sCD4-resistant viruses in the CC1/85 isolate. These assumed relationships should be noted when interpreting later experiments.

### Increased sensitivity of CC101.19 cl.7, but not D1/85.16 cl.23, to CD4i MAbs

**Figure 6:**
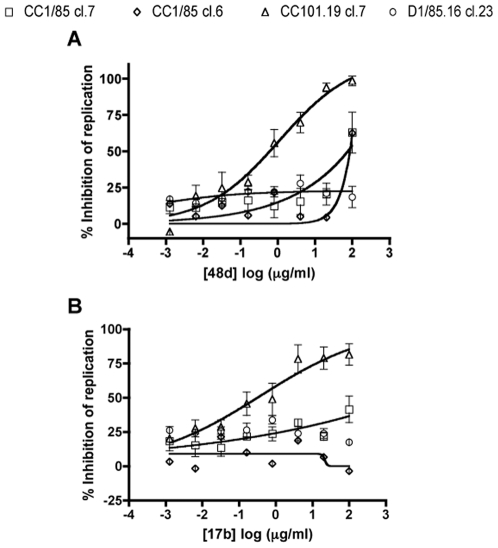
Sensitivity of parental and VVC-resistant clones to MAbs against the CD4i epitope cluster. Chimeric molecular clones CC1/85 cl.7 (squares), CC1/85 cl.6 (diamonds), CC101.19 cl.7 (triangles) and D1/85.16 cl.23 (circles) were used to infect PBMCs in the presence of the indicated concentrations of (A) MAb 48d or (B) MAb 17b. The data represent the extent of inhibition of replication (p24 antigen production) relative to that in the absence of inhibitor (100% replication, 0% inhibition). The data points are mean values±SEM from 3 independent experiments.

MAbs in the CD4i family bind to a CD4-induced epitope on gp120 that substantially overlaps the element of the CCR5 binding site that is located within the bridging sheet and the base of V3. Their interaction with gp120 mimics that of the critical sulfated tyrosine residues in the CCR5 NT [10],[41]. CC101.19 cl.7 was markedly more sensitive than CC1/85 cl.7, CC1/85 cl.6 and D1/85.16 cl.23 to neutralization by CD4i MAbs 48d and 17b (Fig. 6A,B), and also by MAbs ED10, 2.1C and 3.1H against the same epitope cluster (data not shown). Among those five CD4i MAbs, only 48d had even limited neutralizing activity against the two parental clones, and none of them had any detectable activity against D1/85.16 cl.23 (Fig. 6, and data not shown). None of the CD4i MAbs had detectable neutralizing activity against any of the uncloned parental or VVC-resistant isolates (IC_50_ values >100 µg/ml) (data not shown).

In a gp120-capture ELISA, D1/85.16 cl.23 and CC1/85 cl.6 gp120s were the most reactive with MAb 17b (Fig. 5C), which is in marked contrast to the infection-inhibition experiments where D1/85.16 cl.23 and CC1/85 cl.6 were the clones least sensitive to 17b and the related 48d MAb (Fig. 6A,B). Hence although the 17b epitope is well exposed on the gp120 monomer from these VVC-resistant viruses, that exposure is not relevant to what happens with the infectious virus. In the presence of sCD4, 17b bound almost equally well to all four gp120 monomers (Fig. 5C). sCD4 therefore has only a small inductive effect on the 17b epitope on the D1/85.16 cl.23 and CC1/85 cl.6 gp120s, but a much more marked action on gp120s from CC1/85 cl.7 and CC101.19 cl.7 (Fig. 5C). BMS-806 partially inhibited 17b binding to all four gp120s, but its blocking activity was less efficient with CC101.19 cl.7 gp120 than with the other three (Fig. 5D).

The significantly greater sensitivity to CD4i MAbs of CC101.19 cl.7 compared to CC1/85 cl.7 stands in marked contrast to the similar sCD4 sensitivities of these two clones (compare Fig. 4A and Fig. 6). The increased sensitivity of CC101.19 cl.7 to CD4i MAbs may, therefore, be informative about how this clone is VVC-resistant. The simplest explanation is that at least one major element of its CCR5 binding site has become accessible or has been formed constitutively on the native Env complex, and not just after CD4 has bound.

### Differential inhibition of the VVC-resistant viruses by a small molecule V3 ligand

**Figure 7:**
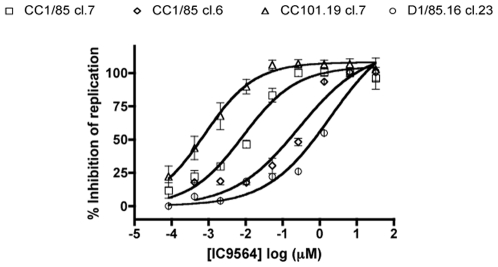
Sensitivity of parental and VVC-resistant clones to the small molecule, V3-targeted inhibitor IC9564. Chimeric molecular clones CC1/85 cl.7 (squares), CC1/85 cl.6 (diamonds), CC101.19 cl.7 (triangles) and D1/85.16 cl.23 (circles) were used to infect PBMCs in the presence of the indicated concentrations of IC9564. The data represent the extent of inhibition of replication (p24 antigen production) relative to that in the absence of inhibitor (100% replication, 0% inhibition). The data points are mean values±SEM from 3 independent experiments.

IC9564 is a small molecule that binds to positively charged residues on the N-terminal side of the V3 stem and/or tip [42],[43]. It does not inhibit CD4 binding or CD4-induced conformational changes, but impedes further structural changes in gp120 that are necessary for fusion, perhaps by locking gp120 in a CD4-induced conformation [43],[44]. CC101.19 cl.7 was the most sensitive of the four clones to IC9564, with an IC_50_ value 11-fold lower than CC1/85 cl.7 (0.62 nM compared to 7.2 nM, respectively). D1/85.16 cl.23 and CC1/85 cl.6 were both much less sensitive to IC9564, with IC_50_ values of 690 and 150 nM, respectively (Fig. 7, Table 2). The relative resistance (∼1100-fold) of D1/85.16 cl.23 compared to CC101.19 cl.7 was only partially recapitulated by the corresponding isolates, for which there was a 10-fold differential in IC_50_ values (Table 2).

These observations suggest that the V3 binding site for IC9564 is significantly more accessible on CC101.19 cl.7, or the corresponding interaction with CCR5 more easily disrupted, than it is on the related parental clone CC1/85 cl.7. However, the IC9564 binding sites on the Env complexes of D1/85.16 cl.23 and its related parental clone CC1/85 cl.6 are much less exposed, or are less relevant to entry. As the V3 sequences of D1/85.16 cl.23 and CC1/85 cl.6 are identical to that of CC1/85 cl.7, sequence differences elsewhere in Env must be responsible for the ∼100 fold differences in IC9564 sensitivities between the first two and the last (Table 2).

### CC101.19 cl.7 has increased sensitivity to V3 MAbs

**Figure 8:**
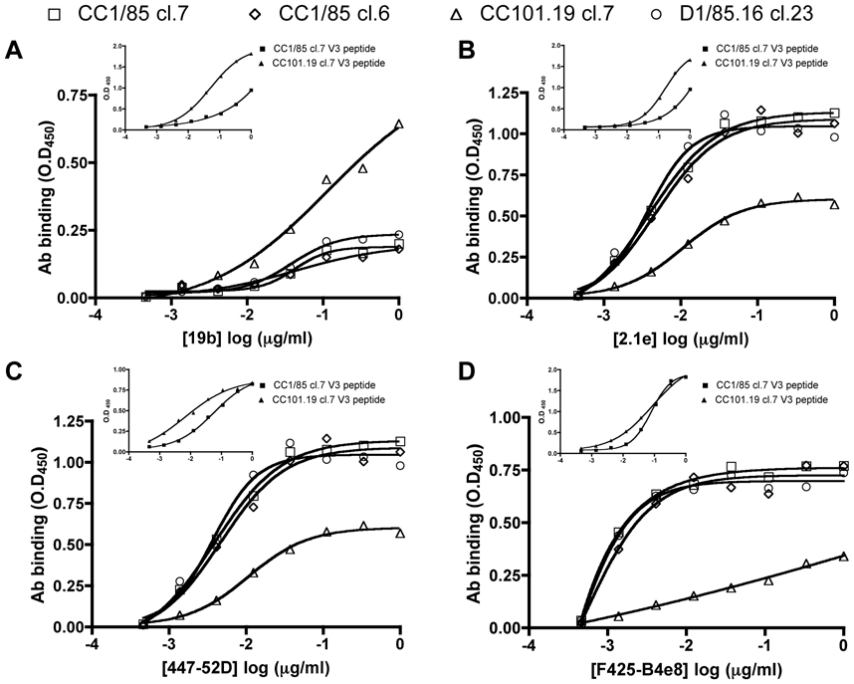
Binding of V3 MAbs to gp120 monomers and V3 peptides. The titration curves represent the binding of gp120 from viral lysates of CC1/85 cl.7 (squares), CC1/85 cl.6 (diamonds), CC101.19 cl.7 (triangles) and D1/85.16 cl.23 (circles) to MAbs (A) 19b; (B) 2.1e; (C) 447-52D; (D) F425-b4e8. MAb binding (OD_450_ values) was corrected for background binding in the absence of gp120. The data points show the results of one representative experiment. The inset panels show the binding of the same MAbs to V3 peptides derived from CC1/85 cl.7 (solid squares) and CC101.19 cl.7 (solid triangles).

The increased sensitivity of CC101.19 cl.7 to IC9564 suggests that its V3 region may also have become more accessible to MAbs. We have shown that several V3 MAbs (447-52D, F425-B4e8 and 39F) lacked significant neutralizing activity against the CC1/85 parental isolate and both VVC-resistant isolates [31]. Since D1/85.16 has the same consensus V3 sequence as CC1/85, this finding suggested that the V3 region of D1/85.16 remained shielded from NAbs, just as it is on most primary isolates. However, the V3 region of CC101.19 contains four sequence changes compared to CC1/85, specifically K305R, H308P, A316V and G321E (Fig. 1C). Variation of this magnitude could directly affect the binding sites for MAbs, limiting their value as probes for V3 accessibility. Indeed, we showed that the four sequence changes destroyed the epitope for V3 MAb 39F on gp120 derived from CC101.19, as assessed by a gp120-capture ELISA [31]. Using the same assay, we found that the V3 epitopes for MAbs F2A3 and C011 were also lost from CC101.19 gp120 compared to CC1/85 cl.7 gp120, and from the corresponding V3 peptide (data not shown). The epitopes for V3 MAbs 19b, 2.1e, 447-52D and F425-B4e8 were, however, still present on CC101.19 cl.7 gp120 (Fig. 8). Indeed, the binding of MAb 19b to gp120 from CC101.19 cl.7 was markedly greater than to the other three gp120s (Fig. 8A). In contrast, although the V3 MAbs 2.1e, 447-52D and F425-B4e8 did bind detectably to CC101.19 cl.7 gp120, they did so to greatly reduced extents compared to the gp120 from the other three viruses (Fig. 8B,C,D). Note that each MAb bound equally well to the gp120s from the two parental clones and D1/85.16 cl.23 (Fig. 8). This observation is consistent with these three gp120s having isogenic V3 sequences (Fig. 1C). We also tested the binding of the MAbs to peptides based on the V3 sequences of CC1/85 cl.7 and CC101.19 cl.7 (Fig. 8, panel insets). For 19b, the peptide-binding and gp120-binding data were consistent, in that the MAb recognized the CC101.19 cl.7 sequences better than CC1/85 cl.7 (Fig. 8A). This was not the case, however, with MAbs 2.1e, 447-52D and F425-B4e8; compared to the CC1/85 cl.7 ligands, 2.1e and 447-52D bound more strongly to the CC101.19 cl.7 peptide but less well to the corresponding gp120, whereas F425-B4e8 bound equally well to both peptides but poorly to CC101.19 cl.7 gp120. Hence the four sequence differences between CC1/85 cl.7 and CC101.19 cl.7 affect the epitopes for different V3 MAbs to different extents when the these epitopes are presented in different contexts (i.e., peptide vs. gp120).

**Figure 9:**
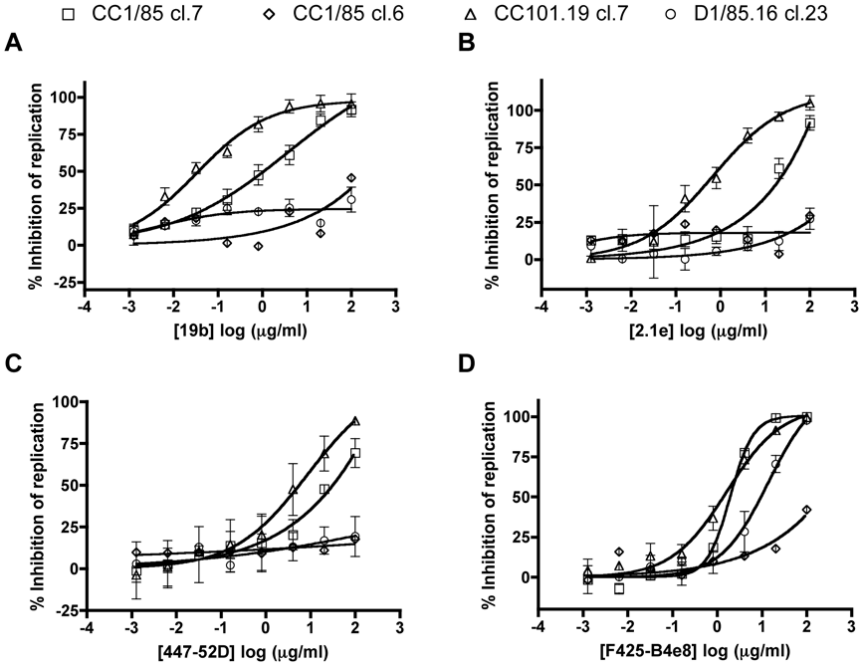
Sensitivity of parental and VVC-resistant clones to V3 MAbs. Chimeric molecular clones CC1/85 cl.7 (squares), CC1/85 cl.6 (diamonds), CC101.19 cl.7 (triangles) and D1/85.16 cl.23 (circles) were used to infect PBMCs in the presence of the indicated concentrations of (A) MAb 19b; (B) MAb 2.1e; (C) MAb 447-52D; (D) MAb F425-B4e8. The data represent the extent of inhibition of replication (p24 antigen production) relative to that in the absence of inhibitor (100% replication, 0% inhibition). The data points are mean values±SEM from 3 independent experiments.

We therefore tested the neutralization activity of V3 MAbs 19b, 2.1e, 447-52D and F425-B4e8 against the four clonal infectious viruses. The resulting data pattern for three of the MAbs was similar to that observed using IC9564. Thus, CC101.19 cl.7 was markedly the most sensitive of the four clones to MAbs 19b, 2.1e and 447-52D (Fig. 9A,B,C; Table 3). Compared to the related parental clone CC1/85 cl.7, the IC_50_ differentials ranged from ∼6-fold for 447-52D to ∼30-fold for 19b and 40-fold for 2.1e (Table 3). However, CC101.19 cl.7 was no more sensitive than CC1/85 cl.7 to MAb F425-B4e8, with an IC_50_ differential of <2-fold (Fig. 9D, Table 3). The increased neutralization sensitivity of CC101.19 cl.7 to 2.1e and, to a lesser extent, 447-52D, was particularly striking given the reduced binding of these MAbs to the corresponding gp120s (compare Figs. 8 and 9). Presumably, the four sequence changes must increase the exposure of the V3 region at the quaternary structural level (i.e., on the CC101.19 cl.7 virus) to an extent that is more than sufficient to overcome any locally adverse impact they may have on the epitope itself (i.e., on gp120). Of note is that the V3 peptide-binding profiles were a better neutralization predictor than the gp120-binding profiles for MAbs 2.1e, 447-52D and F425-B4e8.

**Table 3:** IC_50_ values for inhibition of parental and resistant clones by V3 MAbs.

	CC1/85 cl.7	CC101.19 cl.7	CC1/85 cl.6	D1/85.16 cl.23
19b	1.1±0.40	0.036±0.018	>100	>100
2.1e	17±4.0	0.45±0.058	>100	>100
447-52D	31±1.6	5.5±0.50	>100	>100
F425-B4e8	1.9±0.37	1.4±0.13	>100	9.4±1.0

All IC_50_ values are expressed in µg/ml and are mean of 3 independent experiments±SEM.

In contrast to CC101.19 cl.7, both D1/85.16 cl.23 and the related parental clone CC1/85 cl.6 were highly resistant to V3 MAbs 19b, 2.1e and 447-52D (Fig. 9A,B,C; Table 3). CC1/85 cl.6 was also much more resistant than the other parental clone, CC1/85 cl.7, to neutralization by MAb F425-B4e8, which was the only V3 MAb able to neutralize D1/85.16 cl.23 (Fig. 9D). Given that the V3 sequences of these three clones are identical (Fig. 1C), and that F425-B4e8 binds comparably to all three gp120s (Fig. 8D), quaternary structural differences in the native Env complexes must again be responsible for the neutralization sensitivity differences.

Taken together, the inference of the above experiments is that the V3 region of CC101.19 cl.7 has become unusually accessible to antibodies and a small molecule ligand, even compared to CC1/85 cl.7. In contrast, the V3 region is poorly exposed on CC1/85 cl.6 and on D1/85.16 cl.23, with the exception that the F425-B4e8 epitope is accessible on the latter virus. The two VVC-resistant viruses have therefore taken routes to resistance that not only differ at the genetic level, but also at the phenotypic.

### The V3 region of CC101.19 contains neo-epitopes for NAbs

To create additional antibody probes for studying CC101.19 cl.7, we immunized rabbits (two per group) with 34-residue V3 peptides derived from this virus and also from CC1/85 cl.7, which has the same V3 sequence as CC1/85 cl.6 and D1/85.16 cl.23 (Supporting Information, Text S1). Both V3 peptides were immunogenic in rabbits, inducing antibodies that bound to the cognate and, to a lesser extent, non-cognate, peptide and gp120 in ELISA (Supporting Information; Figs. S1 and S2).

**Figure 10:**
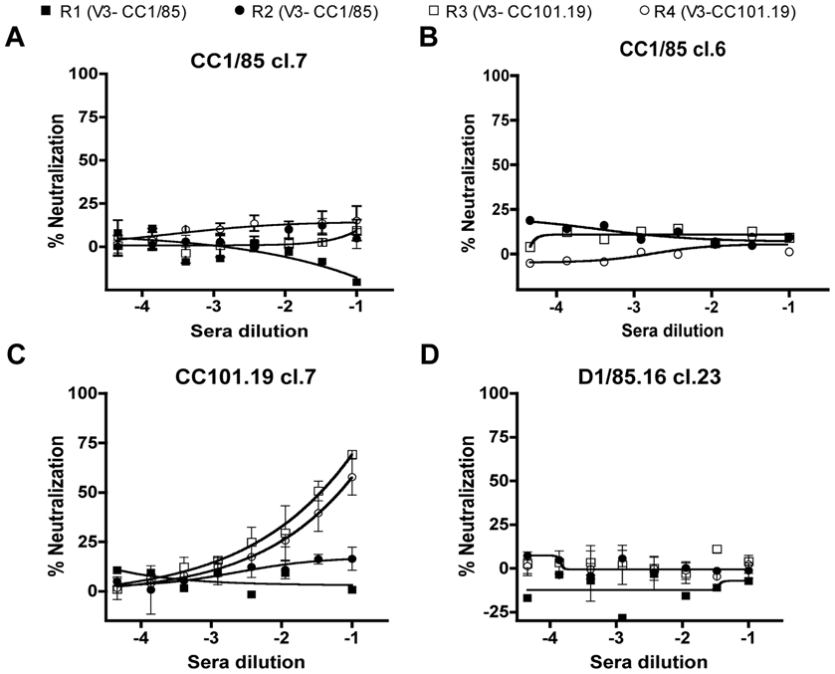
Neutralization of parental and VVC-resistant clones by rabbit anti-V3 peptide sera. Anti-peptide sera (day 49) from rabbits R1 (V3-CC1/85; closed squares), R2 (V3-CC1/85; closed circles), R3 (V3-CC101.19; open squares) and R4 (V3-CC101.19; open circles) were tested for their neutralizing activity against entry of Env-pseudotyped viruses (A) CC1/85 cl.7; (B) CC1/85 cl. 6; (C) CC101.19 cl.7; (D) D1/85 cl.23 into U87-CD4-CCR5 cells. The percent neutralization by immune sera was normalized to the effect of the pre-immune sera at the corresponding dilution. In most case, the effect of pre-immune sera on viral entry was negligible. The values shown are the means±SEM from two independent experiments.

The rabbit anti-V3 sera were then tested for neutralizing activity against the Env-pseudotyped clonal viruses in U87-CD4-CCR5 cells. None of the four antisera neutralized CC1/85 cl.7, CC1/85 cl.6 or D1/85.16 cl.23 (Fig. 10). However, CC101.19 cl.7 was specifically neutralized by the two antisera raised against the autologous V3 peptide (Fig. 10C). Hence the V3 sequence changes that drive VVC resistance have not only caused the V3 region of the CC101.19 Env complex to become more accessible to neutralizing antibodies, they have also created a neo-epitope for the induction of such antibodies.

### Depicting the V3 sequence changes on the V3 structure

**Figure 11:**
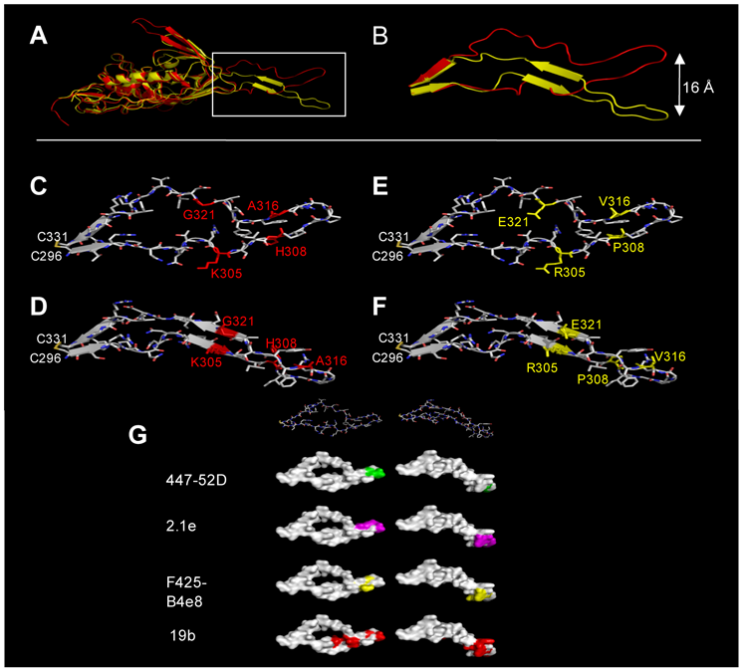
Mapping sequence changes on V3 structures. (A) The CC101.19 cl.7 and CC1/85 cl.7 gp120 structures were modelled with Swiss-Model (http://swissmodel.expasy.org) using template 1 (2B4C.pdb; red) or template 2 (2QAD.pdb; yellow) and superimposed using Pymol (http://www.pymol.org) with a root-mean-square deviation (RMSD) value of 0.892. All the V3 conformations illustrated in this figure are derived from the CD4-bound form of gp120, and differ from the conformation of the unligated form. The box in (A) encloses the V3 region, which is shown in more detail in (B). The residues that differ between the two sequences are colored red (CC1/85 cl.7) or yellow (CC101.19 cl.7) in the V3 models derived using template 1 (C and E) and template 2 (D and F). Note that, in template 2 (F), residues Arg-305 and Glu-321 are positioned close enough together to form a strand-stabilizing salt-bridge. (G) Depiction of the MAb epitopes on the V3 surface. Key contact residues are colored as follows: 447-52D: residues 312, 313 and 315 (green) [48]; 2.1e: residues 313, 314, 315, 316 and 317 (magenta) [49]; F425-B4e8: 309, 315 and 317 (yellow) [50]; 19b: residues 304, 307, 313, 315, 317 and 318 (red) [50]. The V3 templates and models used were the same as shown in Fig. 11 (C,D) and are based on the CC1/85 cl.7 sequence; basing them instead on the CC101.19 cl.7 sequence was not visually informative and the outcome is not depicted.

To assess how the V3 sequence differences between CC1/85 cl.7 and CC101.19 cl.7 may affect the tertiary structure of V3 in the context of gp120, we introduced the two gp120 sequences into two different X-ray crystal structures of a V3-containing gp120 core [9],[10],[43], and then superimposed the resulting models (colored red and yellow, respectively in Fig. 11A,B). In the first template, gp120 is bound to sCD4 and MAb X5 that, like 17b and 48d, binds to the CD4i epitope cluster overlapping the CCR5 binding site [9]. The second template was based on a gp120 core bound to both sCD4 and the tyrosine-sulfated 412 MAb that mimics the CCR5 NT [10]. We elected to use both templates, because unlike template 1, template 2 may mimic the interaction with the tyrosine-sulfated CCR5 NT. The comparison might be informative for understanding why CC101.19 cl.7 has become more dependent on the latter interaction.

Although the gp120 structures align well, the V3 domains assume different structures in the two templates (Fig. 11A,B). In template 1, V3 protrudes from the gp120 core and has three distinct structural regions: (i) a conserved base connected by a disulfide bridge. This β-sheet is part of a 6-stranded β-barrel that forms the core of the gp120 outer domain [45]; (ii) a flexible stem that extends away from the core; and (iii) a β-turn tip (Fig. 11B). In template 2, the tyrosine-sulfated residues bind to the bridging sheet-V3 interface and induce a structural rearrangement in V3 (Fig. 11B). As a result, the N- and C-terminal constituents of the V3 stem are brought into proximity to form a 2-stranded β-sheet that replaces the unstructured V3 stem from the first template. In addition, the V3 tip is displaced by 16 Å (Fig. 11B).

We then inspected where the V3 amino acid changes between CC1/85 cl.7 and CC101.19 cl.7 were located on the two templates (Fig. 11D–F). Two of the substitutions in CC101.19 cl.7, K305R and G321E, are on opposite strands of the β-sheet that is present in the gp120 complex with the tyrosine-sulfated 412d MAb (template 2) but not in the X5 complex (template 1). Both these changes increase the local propensity for forming a β-sheet (Gly, in particular, is accommodated poorly in β-sheets). Moreover, the E321 and R305 side chains in the CC101.19 cl.7 V3 protrude from the same lateral side of the β-sheet and are positioned close enough to form a salt-bridge that could contribute to inter-strand stability. Although the model was based on a V3 conformation derived from a CD4-bound gp120 model, it is possible that a salt bridge could form between E321 and R305, prior to gp120 engagement with CD4 or CCR5. Circular dichroism experiments suggest that a CC101.19 cl.7 V3 peptide has more secondary structure than the corresponding peptide from CC1/85 cl.7 (our unpublished observations). Moreover, and as noted previously, the H308P may facilitate a bend in the V3 structure of CC101.19 cl.7 [20], which may contribute to a relocation of the V3 tip and affect its ability to interact with CCR5.

Thus, the characteristics of the amino acid changes and the available structural data are consistent with a model, based on template 2, in which the CC101.19 cl.7 V3 region constitutively assumes a stabilized conformation that is compatible with binding to the Tyr-sulfated CCR5 NT. In this conformation, the V3 region is more structured, accommodating the binding of the tyrosine-sulfate moieties while at the same time displacing its V3 tip away from the CCR5 ECL2. Whether this model is valid is the subject of ongoing experimental and structural studies.

### Mapping V3 MAb epitopes on the V3 structure

To assist the interpretation of the V3 MAb binding experiments, we mapped the epitopes for MAbs 447-52D, 2.1e, F425-B4e8 and 19b on the V3 crystal structures represented by templates 1 and 2 (Fig. 11G). Note that the available crystal structures for 447-52D and F425-B4e8 with their peptide V3 epitopes reveal more contact residues than are indicated here [46],[47]. However, we choose to focus on the more essential residues revealed by phenotypic analyses [48]–[50]. MAbs 447-52D and 2.1e bind primarily to the V3 tip, although their requirements are subtly different. The essential residues for F425-B4e8 are immediately adjacent to the tip, while 19b also requires residues in the stem. If our interpretation of Fig. 11 is correct, MAbs 19b, 447-52D and 2.1e, but not F425-B4e8, may preferentially recognize the V3 configuration represented by the right-hand panels in Fig. 11G.

## Discussion

Our goal in this study was to learn more about how HIV-1 Env interacts with the CCR5 co-receptor. We used two different but genetically related viruses, CC101.19 and D1/85.16, which have become resistant to small molecule CCR5 inhibitors such as VCV, AD101 and maraviroc. Both resistant variants still use CCR5 for entry, but they have acquired the ability to recognize the inhibitor-CCR5 complex as well as the free co-receptor; their parental strain, CC1/85, can only use free CCR5 for entry and is, therefore, sensitive to small molecule CCR5 inhibitors [18]. Of note is that although both variants share the resistance phenotype, they have taken different genetic routes to it; thus CC101.19 has four amino acid changes in the V3 region of gp120 whereas D1/85.16 has three substitutions in the gp41 fusion peptide [20],[22]. Additional changes elsewhere in Env may contribute to the replication capacity of each virus, but they are neither necessary nor sufficient for resistance [20],[22]. We are now assessing whether these other sequence changes have any influence on any of the phenotypes described here. By studying how these two viruses accomplished the same task in such radically different ways, we reasoned that we might learn something useful about inter-domain interactions within the HIV-1 Env complex. For example, do changes in the fusion peptide have the same effect on Env topology as ones in the V3 region of an entirely different subunit?

We used infectious chimeric viruses and Env-pseudotyped viruses based on clones from the parental and each resistant isolate. The CC1/85 parental isolate was derived from an HIV-1 infected individual who had been infected for at least five years [51],[52]. Accordingly, CC1/85 contains diverse quasispecies. Here, we studied two different clones (cl.6 and cl.7) derived from the CC1/85 isolate, and one clone from each resistant isolate, i.e. CC101.19 cl.7 and D1/85.16 cl.23. The phenotypic properties of these clones are summarized in Table S1. A multiple sequence alignment analysis showed that the amino-acid sequence of CC1/85 cl.6 is more related to that of D1/85.16 cl.23 whereas CC1/85 cl.7 more resembles CC101.19 cl.7, although it is not possible to prove evolutionary relationships. Of note is that CC1/85 cl.7 is much more sensitive than CC1/85 cl.6 to sCD4, the V3-targeting compound IC9564 and NAbs against V3. Thus, CC1/85 cl.7 behaves more like a T-cell line-adapted virus than a primary virus in this regard. The genetic determinants of this clone's sensitivity to NAbs and CD4-targeted inhibitors must lie outside its V3 region, which is identical to those of the more resistant clones CC1/85 cl.6 and D1/85.16 cl.23.

This atypical phenotype of CC1/85 cl.7 underlies its use as a comparator virus for CC101.19 cl.7, which is also unusually sCD4-sensitive. Although we cannot exclude the possibility that this property of CC101.19 cl.7 is relevant to its VVC-resistance phenotype, we strongly suspect otherwise. Instead, we think that CC101.19 cl.7 evolved from a sCD4-sensitive parental virus that shares certain Env characteristics with CC1/85 cl.7. The parental virus for D1/85.16 cl.23 was, in contrast, probably one of the more prevalent sCD4-resistant variants that resemble CC1/85 cl.6. Passage of HIV-1 primary isolates creates sCD4 sensitivity not only in T-cell lines [34],[53] but also in PBMC [32]. Thus, when the CC1/85 parental isolate was cultured in primary CD4^+^ T cells for 19 passages in the absence of any selecting compound, as a control for AD101-selection pressure, the resulting CCcon.19 isolate was substantially more sensitive to sCD4 and MAb b12, but not to several other MAbs and inhibitors that target other Env regions [32]. The genetic determinants of the sCD4 sensitivity of CCcon.19 lay within the gp120 V2 loop: substitutions I165K and D167N, and an SN deletion at positions 188–189 [32]. Clones CC1/85 cl.7, CC101.19 cl.7 and D1/85.16 cl.23 all have the D167N substitution, but CC1/85 cl.6 does not, while the I165K substitution and the SN deletion at positions 188–189 were absent from all four clones (data now shown). The variation at residue 167 is sufficient to partially explain the marked difference in sCD4 sensitivity between the two parental clones [32]. Most of the amino acid differences between the comparator pairs (CC1/85 cl.7 vs. CC101.19 cl.7 and CC1/85 cl.6 vs. D1/85 cl.23) lie within the V4 and V5 regions (Fig. 1B). Of note is that the differences in sCD4 sensitivity between the various parental and resistant clones were not attributable to variation in binding of the gp120 monomer to CD4. Thus, as is usually the case, the efficiency of sCD4 neutralization is determined by the quaternary structure of the native Env complex [33],[36],[37].

The BMS-806 sensitivity profiles of the parental and resistant clones (and isolates) were the inverse of those seen using sCD4, which is consistent with a previous report [54]. Thus D1/85.16 cl.23 was simultaneously the clone most resistant to sCD4 but the most sensitive to BMS-806, and conversely for CC101.19 cl.7. However, as seen with sCD4, BMS-806 sensitivity also varied markedly between the two parental clones; CC1/85 cl.6 behaved akin to D1/85.16 cl.23 while CC1/85 cl.7 again resembled CC101.19 cl.7. Moreover, the pattern of infection-inhibition data for BMS-806 and the four clones was reflected in the outcome of a gp120-CD4 inhibition assay using the corresponding monomeric gp120s. Hence, as with sCD4 sensitivity, we do not believe the different responses of the various clones to BMS-806 are causally related to CCR5 inhibitor resistance. The increased BMS-806 sensitivity of CC1/85 cl.6 and D1/85.16 cl.23 compared to the other two clones may simply reflect how gp120 sequence variation affects BMS-806 binding, although none of the four clones contained any amino acid changes known to be associated with BMS-806 resistance [55].

The current model of how gp120 interacts with CCR5 posits that two different structural elements of each protein are involved: the tip of V3 region of gp120 binds to ECL2 of CCR5, the base and stem of V3 and the bridging sheet to the NT [8]–[12]. To allow multi-point attachment, the twin-elements of each protein must be folded into an appropriate geometry. It seems a reasonable assumption that the binding of a small molecule inhibitor alters the orientation between the ECL2 and NT regions, disrupting the multi-point binding site for gp120 and thereby impeding the gp120-CCR5 interaction. Hence the simplest hypothesis for how the resistant viruses recognize both the inhibitor-bound and –free forms of CCR5 is that they use a single-point attachment mechanism. In other words, their Env complex interacts with either ECL2 or the NT, and does so in a way that is unaffected, or little affected, by a small molecule CCR5 inhibitor. Our experiments with CCR5 point-mutants were designed to test this hypothesis. We observed that the two escape mutants do indeed differ in their usage of ECL2 and the NT, when compared both to each other and to parental clones. Thus, CC101.19 cl.7 was highly dependent on residues in the CCR5 NT, namely Y10, Y14 and C20; in contrast, D1/85.16 cl.23 was markedly less affected by changes in the NT but was slightly more sensitive to some ECL2 substitutions (Table 1). It is also noteworthy that CC101.19 cl.7 and CC1/85 cl.7 were comparably sensitive to some ECL2 mutations. Overall, both resistant viruses were somewhat less tolerant of CCR5 mutations than their comparator clones, which may reflect their acquired capacity to also use the inhibitor-CCR5 complex. That constraint might reduce the robustness and promiscuity of the normally rather plastic Env-CCR5 interaction; we have hypothesized that resistant viruses preferentially use certain conformational subspecies or isoforms of both free and inhibitor-complexed CCR5 [22].

We next used MAbs and a small molecule that interact with the different elements of the CCR5 binding site on gp120, as additional probes of differences in the CCR5 interactions of the two resistant clones. The VVC-resistant clone CC101.19 cl.7 was markedly the most sensitive of the four clones to five different MAbs against the CD4i epitope cluster on gp120 that is an important element of the CCR5 binding site, the bridging sheet. The sensitivity of CC101.19 cl.7 to CD4i MAbs was significantly greater than its comparator parental clone CC1/85 cl.7, suggesting it was relevant to the VVC-resistance phenotype and not just a general property of this particular clonal lineage. Of note is that the binding of CD4i MAbs to the various gp120 proteins was not correlated with how the same MAbs neutralized the corresponding viruses. Hence the increased neutralization sensitivity of CC101.19 cl.7 to CD4i MAbs must be determined by the quaternary structure of the native Env trimer, not the topology of the gp120 monomer. Overall, we suggest that at least one major element of the CCR5 binding site, the CD4i epitope cluster, has become constitutively exposed on the native Env complex of CC101.19 cl.7. Alternatively, the geometry of the interaction between the mutant Env complex and the target cell surface may be one in which the normal steric constraint on the binding of CD4i NAbs has become relaxed. However, D1/85.16 cl.23 remains highly resistant to CD4i MAbs, irrespective of whether they are tyrosine-sulfated (47e, 412d, CM51 and E51) or not (48d and 17b), implying that the fusion peptide changes and the V3 changes affect Env topology differently.

The IC9564 small molecule binds to positively charged residues in the V3 stem, N-terminal to the tip. It competes with MAbs 39F and 447-52D that target the same region [42]. However, IC9564 resistance is associated with amino acid changes in the bridging sheet [44]. The binding of IC9564 to V3 has been proposed to lock gp120 into a CD4-induced conformation in which CD4i epitopes become more exposed [43]. We observed that CC101.19 cl.7 was the most sensitive of the four clones to IC9564, again significantly more so than the comparator parental clone, CC1/85 cl.7. Hence either the V3 binding site for IC9564 is significantly more accessible on CC101.19 cl.7 than on CC1/85 cl.7, or the interaction between the V3 region of CC101.19 cl.7 and the CCR5 NT is more vulnerable to disruption by the small molecule ligand. In comparison, D1/85.16 cl.23 and CC1/85 cl.6 were highly resistant to IC9564. Because the V3 sequences of D1/85.16 cl.23 and CC1/85 cl.7 are identical, sequence changes elsewhere in Env must underlie the ∼100-fold difference in their IC9564 sensitivities, presumably by affecting how well its V3 binding site is exposed on the Env complex.

Similar results were obtained using several MAbs to the V3 region of the four clones. Again, CC101.19 cl.7 was markedly the most sensitive virus, even compared to CC1/85 cl.7. This is particularly remarkable given that the four sequence changes in its region V3 actually reduce the binding of some of the MAbs to the corresponding gp120 monomer (and destroy the epitopes for other V3 MAbs). Taken together with the data on IC9564 sensitivity, these observations suggest that the V3 region of CC101.19 cl.7 is well exposed on the native Env complex. But, as with the CD4i epitope cluster, this is not the case with D1/85.16 cl.23; the V3 region of this clone remains sequestered, perhaps even more so than on the comparator parental clone. The increased exposure of V3 on CC101.19 cl.7 Env presumably facilitates interactions with the CCR5 NT, but a side effect is to render the virus highly sensitive to NAbs that target V3. Other studies have also shown that deletions or other radical changes in V3 disrupt the interaction between this region of gp120 and ECL2 of CCR5, and thereby render HIV-1 more dependent on the CCR5 NT for binding and entry [56],[57].

In summary, we propose that the structural change created by the four amino acid substitutions in CC101.19 cl.7 impairs the interaction of the V3 tip with ECL-2, while promoting the binding of the V3 base and bridging sheet to the NT; the latter outcome would account for the increased dependence of CC101.19 cl.7 on sulfated tyrosine residues 10 and 14 and its enhanced sensitivity to MAbs that bind to CD4i epitopes associated with the bridging sheet. We have depicted the four sequence changes on the conformation of V3 in the context of the gp120 monomer, using structural templates that may represent the V3 structures that exist before and after binding to the tyrosine-sulfated CCR5 NT. One outcome of the model is that, in the CD4-bound form of gp120 (template 2), the K305R and G321E changes may create an inter-strand salt-bridge that stabilizes this V3 configuration in CC101.19 cl.7 Env and perhaps facilitates its interaction with the CCR5 NT. Furthermore, the H308P change introduces a kink into the V3 region that may affect the geometry of the tip and impair its ability to interact with ECL2. These suppositions are, of course, speculative pending additional structural information.

A similar substitution pattern, R305K+G321E, at the same V3 residues occurred when escape mutants to VVC emerged in a different viral context *in vivo* [13]. The R305K change is the inverse of what happens in CC101.19 cl.7, but preserves a cationic residue at this position; the G321E substitution is identical to the one arising in CC101.19 cl.7 and introduces an anionic amino acid. Perhaps in the different genetic contexts, the two changes together create the same salt bridge and have the same effect of stabilizing a V3 conformation that is better able to interact with the CCR5 NT.

There were differences between the various V3 MAbs in how they neutralized the various clones, and in how they bound to the corresponding gp120s and V3 peptides. Of particular note is that MAb 19b bound markedly better to gp120 and the V3 peptide from CC101.19 cl.7, and neutralized the corresponding virus much more potently, compared to the other gp120s, peptide and viruses. Thus the four sequence changes that created CCR5 inhibitor resistance must have had a substantial effect on the topology of at least part of the V3 region of CC101.19 cl.7 that is apparent at the levels of both the gp120 monomer and a simple V3 peptide. MAbs 2.1e, F425-B4e8 and 447-52D, however, bound less well to CC101.19 cl.7 gp120, but still neutralized the corresponding virus more potently than the comparator clone, CC1/85 cl.7. The IC_50_ differentials between CC101.19 cl.7 and CC1/85 cl.7 also varied from MAb to MAb, being much lower for F425-B4e8 and 447-52D than for 19b and 2.1e. Some sub-regions of V3 may therefore be more exposed than others on the CC101.19 cl.7 Env complex. Consistent with this idea, the epitopes for 19b, 2.1e, 447-52D and F425-B4e8 are located in different regions of V3. The X-ray crystal structure of the 447-52D complex with a V3 peptide shows that the side chains of six amino acid residues upstream of the GPGR turn are arranged into a β-hairpin and form a β-sheet with the side chains of a corresponding strand of the MAb [58]. F425-B4e8, on the other hand, interacts with the V3 crown, particularly with Arg-315 [47]. Of note is that F425-B4e8 was the only V3 MAb able to neutralize D1/85.16 cl.23, so perhaps the region around Arg-315 is the only part of V3 exposed on D1/85.16 cl.23, and hence accessible to F425-B4e8. Structural information is not available for 19b and 2.1e, but mutagenesis and phage display data suggest that 19b recognizes flanking region in V3 upstream to the crown while 2.1e sees elements of the crown and downstream flanking residues [49],[50],[59]. If our interpretation of the V3-gp120 modelling data is correct, then MAbs 19b (in particular), 447-52D and 2.1e, but not F425-B4e8, may preferentially recognize the V3 configuration that is stabilized by the salt bridge between Arg-305 and Glu-321 in CC101.19 cl.7 gp120.

The V3 region of CC101.19 cl.7 is also a neo-epitope, in that a peptide containing the four amino acid changes was immunogenic in rabbits, inducing Abs that were able to neutralize the corresponding virus via its exposed V3 region. We reported previously that both the CC101.19 and D1/85.16 isolates are more sensitive than the parental isolate to various anti-Env MAbs and sera from HIV-1 infected people, albeit usually not to a dramatic extent [31]. The present results confirm and extend those findings. Hence, variants that arise *in vivo* under the selection pressure of maraviroc or VCV might be more vulnerable than wild type viruses to NAbs raised against both existing and neo-epitopes on their Env complexes, particularly the V3 region. In other words, to resist the CCR5 inhibitors, HIV-1 will need to adapt in a way that also preserves its existing defences against humoral immunity. The twin constraints on the Env complex might therefore create variants with interesting and informative properties. We do not yet know, but are investigating, whether the increased exposure of V3 and other neutralization epitopes is obligatorily linked to the sCD4-sensitivity of a subset of parental clones. The parental isolate was derived from a patient with chronic HIV-1 infection in whom X4 viruses were detected a year later and has considerable quasispecies diversity [52]. Its properties may be relevant, given current clinical practice with CCR5 inhibitors and the generation of resistance *in vivo* [60].

Overall, we conclude that the two resistant variants have adapted to the selection pressure of the small molecule CCR5 inhibitors in different ways, both genetically and phenotypically. They are similar in that both can use the inhibitor-CCR5 complex and free CCR5 for entry, but they differ in how they do so. The four V3 sequence changes in CC101.19 cl.7 have constitutively exposed elements of the CCR5 binding site on the native Env complex of this virus, both within V3 and associated with the CD4i epitope cluster. These changes facilitate interactions of the Env complex with the tyrosine-sulfated CCR5 NT. It remains a mystery, however, how the three fusion peptide changes in D1/85.16 cl.23 render this virus CCR5 inhibitor-resistant. The V3 and bridging sheet elements are no more exposed, and perhaps even less well exposed, on D1/85.16 cl.23 than on the comparator parental clone, and there is no compelling evidence from the CCR5 mutant panel as to how the virus-coreceptor interactions differ. We note, however, that even mutations in the gp41 cytoplasmic tail can enhance interactions with CCR5, allowing the mutant virus to infect cells that express only trace amounts of the coreceptor [61]. Perhaps D1/85.16 cl.23 interacts productively only with subpopulations of free and liganded CCR5 that are sometimes available only in limited quantities [22]. The CCR5-triggered conformational changes in Env that drive fusion may have different quantitative and qualitative requirements for the fusion peptide- and cytoplasmic tail-gp41 mutants, compared to wild type viruses. Additional studies will need to be performed to address such concepts and thereby enable us to better understand the D1/85.16 cl.23 resistance mechanism.

## Materials and Methods

### Cells and cell culture

U87-CD4 and U87-CD4-CCR5 cells, contributed by Dr. HongKui Deng and Dr. Dan Littman, were obtained from the NIH AIDS Research and Reference Reagent Program (ARRRP) [62]. 293T cells were from the American Type Culture Collection (ATCC; Manassas, VA). All these cell lines were maintained in Dulbecco's modified Eagle medium (DMEM; Invitrogen, Carlsbad, CA), supplemented with 10% fetal bovine serum (FBS; Invitrogen) and 100 U/ml penicillin+100 µg/ml streptomycin (1× PenStrep; HyClone, Logan, UT) and L-glutamine (Invitrogen).

PBMC were purified from leukopacks obtained from the New York Blood Center (New York, NY) and stimulated as previously described [20]. Briefly, the leukopacks were depleted of CD8^+^ cells using the RosetteSep reagent (StemCell Technologies, Vancouver, BC, Canada) and then purified on a Ficoll density gradient. Cells from each blood donor were split into two cultures, one of which was stimulated for three days with surface-immobilized anti-CD3 MAb (clone OKT3), the other with 5 µg/ml of phytohemagglutinin (PHA; Sigma). The PBMC culture medium was RPMI 1640 (Invitrogen) supplemented with 10% FBS, 1× PenStrep and 100 U/ml of interleukin-2 (IL-2; ARRRP, donated by Hoffmann-La Roche, Inc.). All cells were incubated at 37°C in an atmosphere containing 5% CO_2_.

### Antibodies and compounds

MAbs 17b, 48d, 19b and 2.1e were gifts from Dr. James Robinson (Tulane University, New Orleans, LA), MAb b12 from Dr. Dennis Burton (Scripps Research Institute, La Jolla, CA). RPA-T4 was purchased from Santa Cruz Biotech, CA. The MAbs 447-52D and F425-B4e8 were obtained through NIH AIDS Research and Reference Reagent Program, Division of AIDS, NIAID, NIH, contributed by Dr. S. Zolla-Pazner and Dr. M. Poster, respectively. sCD4 and CD4-IgG2 were donated by Dr. William Olson (Progenics Pharmaceuticals, Tarrytown, NY), BMS-806 by Dr. Richard Colonno (Bristol-Myers Squibb, Wallingford, CT). The gp120-targeting compound IC9564 was provided by Dr. Chin-Ho Chen (Meharry Medical College, Nashville, TN). The small molecule CCR5 inhibitor VVC (SCH-D, SCH-417690) [63] was provided by Dr. Julie Strizki (Schering-Plough Research Institute, Kenilworth, NJ).

### Viruses, gp120 proteins and CCR5 mutants

The construction of the pNLluc-AM and PCI-Env plasmids has been previously described [18]. In brief, the pNLluc-AM vector consists of the pNL4-3 proviral plasmid, in which a portion of the *env* gene was deleted and replaced with an SV40 promoter/firefly luciferase cassette using a yeast recombination system [64]. The pCI-env expression plasmids were constructed by insertion of the CC1/85 cl.7, CC1/85 cl.6, CC101.19 cl.7 and D1/85.16 cl.23 *env* genes into the multiple cloning site of pCI (Promega, Madison, WI) at the EcoRI-XhoI restriction site. The construction and properties of clonal viruses pNL4-3/env derived from CC1/85 cl.7, CC1/85 cl.6, CC101.19 cl.7 and D1/85.16 cl.23 have been previously described [14],[18],[20].

The PPI4-CC1/85 cl.7 and PPI4-CC101.19 cl.7 gp120 expression plasmids were cloned as previously described [20]. Briefly, KpnI-BbvCI fragments from the desired *env* gene were subcloned into the pPPI4-JR-FL gp140 vector [65]. Two consecutive in-frame stop codons were then introduced by QuickChange mutagenesis (Stratagene), immediately following the lysine in the sequence REKR, the natural cleavage site between gp120 and gp41.

All CCR5 mutants were provided by Dr. Tanya Dragic (Albert Einstein College of Medicine, Bronx, NY) except for Y10A, Y14F and Y14Q, which were donated by Dr. David Kabat (Oregon Health and Science University, Portland, OR).

### Virus and pseudovirus preparation

pNL4-3/*env* plasmids were constructed as previously described [20],[22]. Infectious clonal virus stocks were prepared by transient transfection of 293T cells with pNL4-3/*env* plasmids using Lipofectamine 2000 (Invitrogen, Carlsbad, CA) according to the manufacturer's instructions, as described elsewhere [20]. All stocks of infectious viruses were passed through a 0.45-µm filter and stored in aliquots at −80°C. The titers (50% tissue culture infectious dose; TCID_50_) of all stocks were determined in PBMC culture by standard methods [66].

Env-pseudotyped viruses were made by co-transfecting 293T cells with a 3∶1 ratio of the plasmids pCI-*env* and pNLluc-AM, using Lipofectamine 2000 (Invitrogen) according to the manufacturer's instructions. One day after transfection, the cells were washed with culture media and incubated for one additional day. The virus-containing supernatants were passed through a 0.45-µm filter immediately before use.

### Sequence analysis

To determine similarities between amino acid sequences, a Clustal W multiple sequence alignment (MSA) of Env amino acid sequences was generated using MacVector 10.0.2. Env sequences have been previously deposited in GenBank (accession numbers AY35338 through AY357345, AY357465 and FJ713453) [20],[22].

### HIV-1 infection of PBMC

The sensitivity of the infectious viral clones to gp120-targeting MAbs and other inhibitors was assessed as previously described [18],[20]. Briefly, 2×10^5^ PBMC were seeded into each well of a 96-well culture plate after 3 days of stimulation. The PBMC consisted of equal numbers of cells from each of the two stimulation conditions outlined above, and were derived from two individuals. The viral clones (at 100 TCID_50_) were incubated with the same volume of culture media containing twice the desired concentration of the inhibitor (IC9564, sCD4, BMS-806) or MAb for 1 h at 37°C. After this incubation, 100 µl of the virus-inhibitor mixture were added to 100 µl of PBMCs. Production of the HIV-1 p24 antigen after 7 days of culture was quantified using an in-house ELISA [67]. Entry inhibition in the presence of MAbs or V3-targeting compounds was calculated as 100×[1−(p24_MAb_/p24_control_)], the control being infection in the absence of an inhibitor or MAb. Titration curves were generated using Prism (Graphpad software, San Diego, CA) and used to determine the IC_50_ values.

### HIV-1 infection of U87-CD4 cells expressing CCR5 mutants

U87-CD4 cells were transfected with CCR5-expressing plasmids using lipofectamine 2000 (Invitrogen) according to the manufacturer's instructions. One day after transfection, the cells were washed twice with culture media.

One aliquot of cells was seeded into a 6-well plate and used for determination of CCR5 expression by FACS on the following day. Different amounts of the CCR5-WT plasmid were transfected and the MFI value for CCR5 expression was calculated under each condition, to generate a dose-response curve. The extent of entry mediated by each mutant CCR5 plasmid was then compared to that mediated by wild-type CCR5 at the same MFI value.

The remaining cells were seeded into 96-well plates at a density of 1×10^4^ cells per well in 100 µl of media for one more day. Then, freshly harvested Env-pseudoviruses were pre-incubated with magnetic beads (ViroMag R/L; OZ Biosciences, Marseille, France) for 15 min, added to the transfected cells at a volume of 100 µl and placed on a Super Magnetic Plate (Oz Biosciences) for 10 min, as recommended by the manufacturer. The cultures were then maintained for 72 h at 37°C. A 100 µl aliquot of culture supernatant was then removed and replaced with 100 µl of Bright-Glo Luciferase Substrate (Promega Inc). After 5 min, the plates were analyzed in a Victor3 1420 plate-reading luminometer (Perkin Elmer, Wellesly, MA). There was no measurable luminescence from uninfected cells.

### HIV-1 infection of U87-CD4-CCR5 cells and inhibition by rabbit sera

For studies with rabbit sera, Env-pseudoviruses were incubated with the same volume of media containing twice the required dilution of sera for 1 h at 37°C, then the mix was added to U87-CD4-CCR5 cells for 72 h before measurement of luciferase expression. The percent neutralization by rabbit sera was calculated as previously described [68]. To correct for any interference from rabbit serum components, a pre-immune serum sample from the same animal was processed identically to the post-immune samples in each experiment, to enable the determination of the percent neutralization at each serum dilution. Percent neutralization was defined as [1−(RLUpostimmune/RLUpreimmune)]×100. The effect of this adjustment was, in most cases, negligible; neutralization titers derived using the pre-immune serum correction were usually very similar to those obtained using the standard control wells, containing only Env-pseudoviruses and cells, as a reference. Non-linear sigmoidal dose-response curves were generated using Prism (Graphpad software, San Diego, CA).

### gp120 capture ELISA

MAb binding to gp120 was quantified essentially as described previously [33],[69]. Supernatants from 293T cells transfected with either PPI4-CC1/85 cl.7 or PPI4-CC101.19 cl.7, or viral lysates served as the sources of gp120; they were added to ELISA wells coated overnight with sheep antibody D7324 to the gp120 C-terminus (Aalto Bioreagents, Rathfarnham, Dublin, Ireland). The plates were washed three times with TSM (20 mM Tris, 150 mM NaCl, 1 mM CaCl_2_, 2 mM MgCl_2_), and then blocked with TSM/1% BSA for 30 min. MAbs diluted in TSM were then added for 2 h. The plates were washed five times with TSM/0.05% Tween, before addition of an appropriate HRP-labeled secondary Ab in TSM/0.05% Tween for 1 h. The colorimetric endpoint at 450 nm was determined 10 min after the addition of the substrate solution (0.1 M sodium acetate, 0.1 M citric acid, 1% TMB (Sigma-Aldrich, St. Louis, MO) and 0.01% H_2_O_2_). Non-linear sigmoidal dose-response curves were generated using Prism (Graphpad software, San Diego, CA).

## Supporting Information

Figure S1 Binding of rabbit anti-V3 sera to V3 peptides. Rabbits were immunized with V3 peptides: (A, B) Rabbits R1 and R2 (V3-CC1/85 sequence); (C, D) rabbits R3 and R4 (V3-CC101.19 sequence). Sera drawn on day 49 were tested for reactivity with the CC1/85 (squares) or CC101.19 (triangles) V3 peptides in an ELISA. The OD_490_ values shown were corrected for background binding, as measured using pre-immune sera. (E) Endpoint and midpoint titers were calculated using Prism Graphpad software.
(0.20 MB PDF)

Figure S2 Binding of rabbit anti-V3 sera to gp120. The titration curves depict the binding of sera from rabbits R1 (V3-CC1/85; closed squares), R2 (V3-CC1/85; closed circles), R3 (V3-CC101.19; open squares) and R4 (V3-CC101.19; open circles) to gp120 from (A) CC1/85 cl.7 or (B) CC101.9 cl.7. The OD_450_ values shown were corrected for background binding, as measured using pre-immune sera.
(0.11 MB PDF)

Table S1 Summary of some phenotypic characteristics of CCR5 inhibitor-sensitive and -resistant viral clones.
(0.07 MB PDF)

Text S1 Supporting results and methods.
(0.09 MB PDF)
