# Supplementary figures and images for: Two HIV-1 Variants Resistant to Small Molecule CCR5 Inhibitors Differ in How They Use CCR5 for Entry
Source: PLoS Pathog. 2009 Aug 14;5(8):e1000548. doi: 10.1371/journal.ppat.1000548 (PMC2718843; doi:10.1371/journal.ppat.1000548)

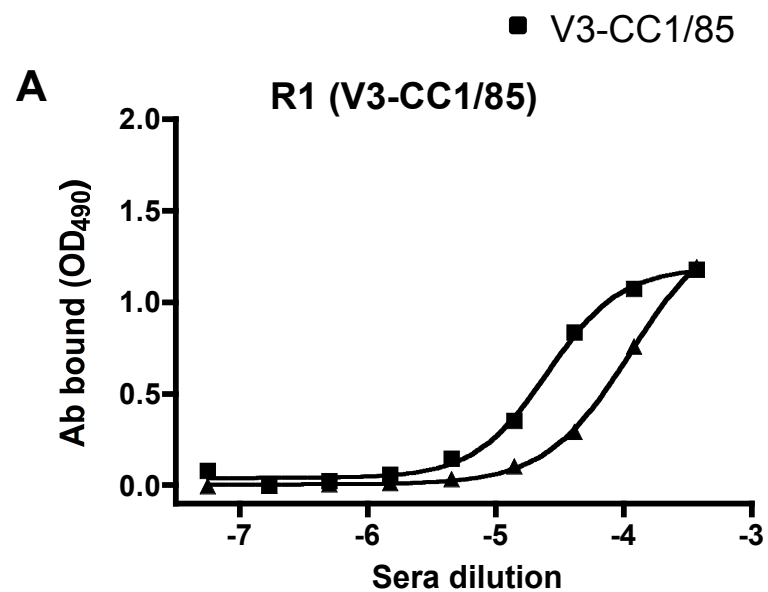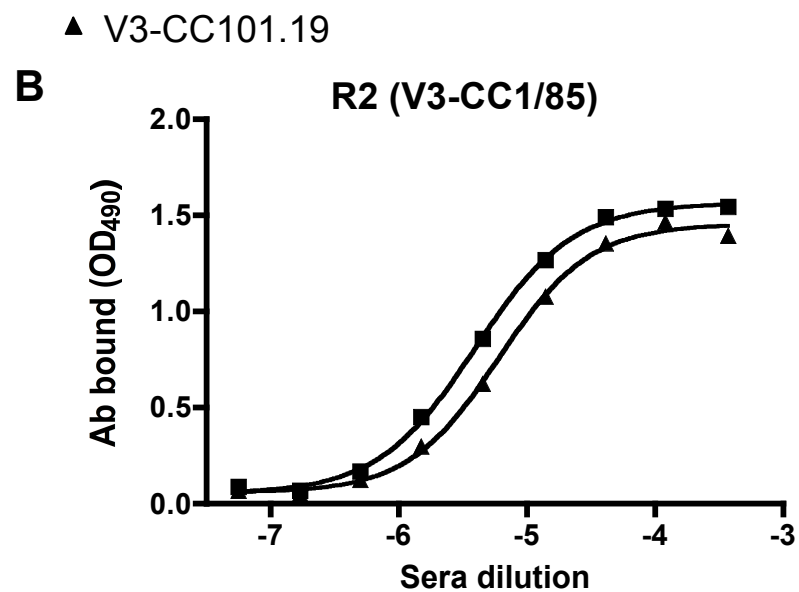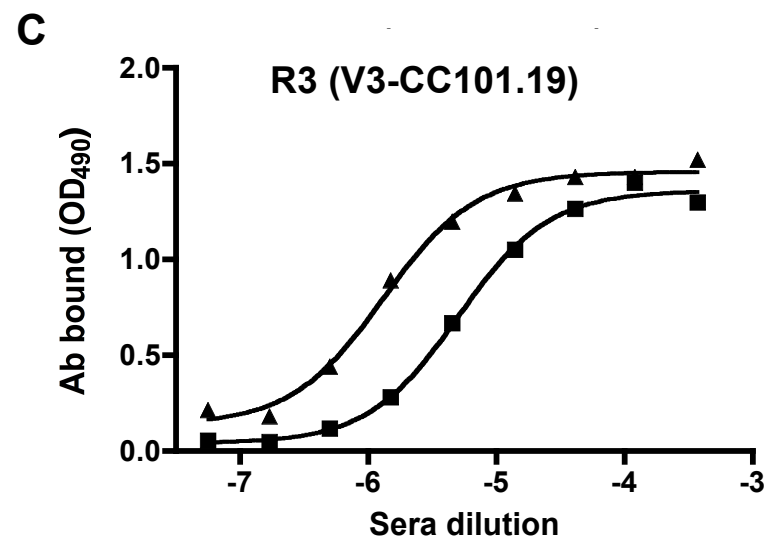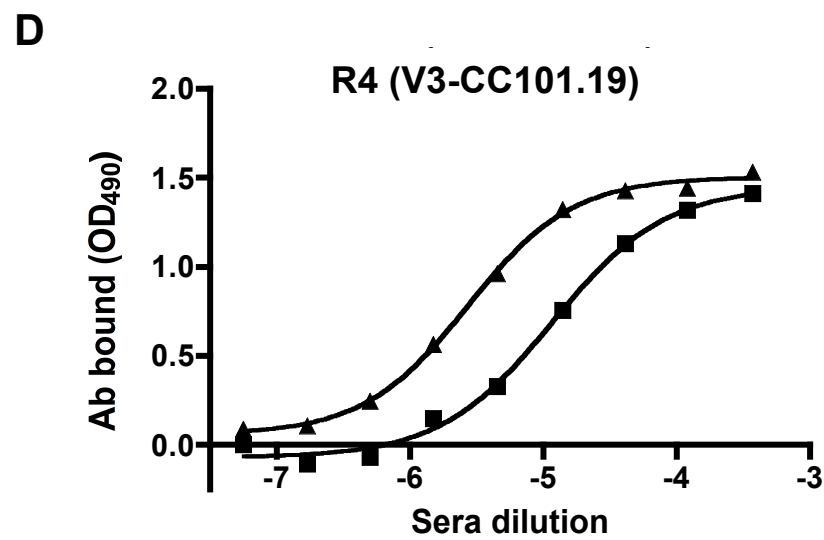

**E**

|                           | V3-CC1/85 |      |      |      | V3-CC101.19 |      |     |     |
|---------------------------|-----------|------|------|------|-------------|------|-----|-----|
|                           | R1        | R2   | R3   | R4   | R1          | R2   | R3  | R4  |
| Endpoint titer ( $10^6$ ) | 0.77      | 24.4 | 3.5  | 2.03 | 0.19        | 8    | 8.4 | 4.6 |
| Midpoint titer ( $10^5$ ) | 0.41      | 2.7  | 2.12 | 0.79 | 0.088       | 1.81 | 9.3 | 4   |

Supplement: Figure S1 — Binding of rabbit anti-V3 sera to V3 peptides. Rabbits were immunized with V3 peptides: (A, B) Rabbits R1 and R2 (V3-CC1/85 sequence); (C, D) rabbits R3 and R4 (V3-CC101.19 sequence). Sera drawn on day 49 were tested for reactivity with the CC1/85 (squares) or CC101.19 (triangles) V3 peptides in an ELISA. The OD490 values shown were corrected for background binding, as measured using pre-immune sera. (E) Endpoint and midpoint titers were calculated using Prism Graphpad software. (0.20 MB PDF) [file ppat.1000548.s001.pdf]

■ R1 (V3- CC1/85)      ● R2 (V3- CC1/85)      □ R3 (V3- CC101.19)      ○ R4 (V3- CC101.19)

**A**

**CC1/85 cl.7 gp120**

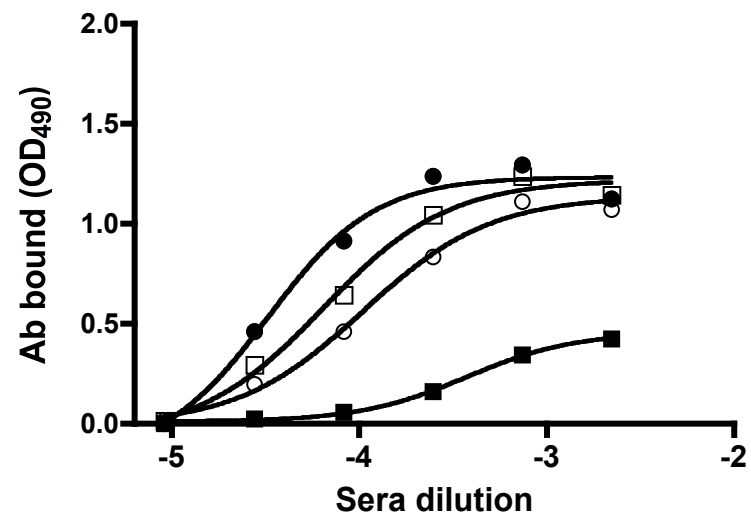

**B**

**CC101.19 cl.7 gp120**

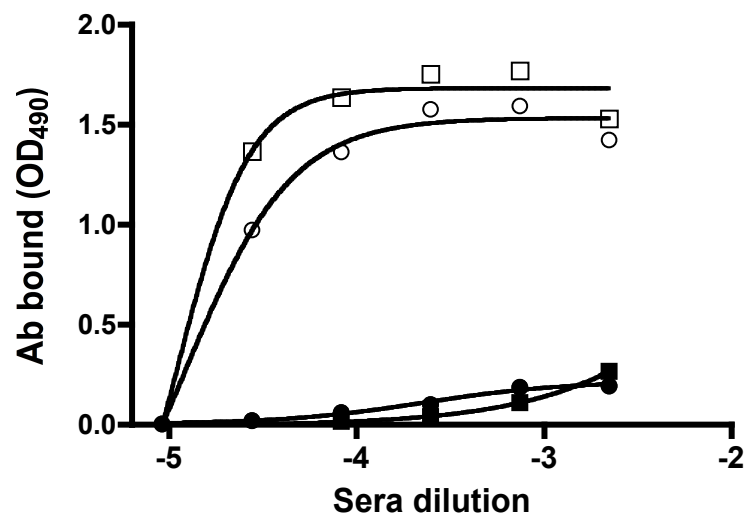

Supplement: Figure S2 — Binding of rabbit anti-V3 sera to gp120. The titration curves depict the binding of sera from rabbits R1 (V3-CC1/85; closed squares), R2 (V3-CC1/85; closed circles), R3 (V3-CC101.19; open squares) and R4 (V3-CC101.19; open circles) to gp120 from (A) CC1/85 cl.7 or (B) CC101.9 cl.7. The OD450 values shown were corrected for background binding, as measured using pre-immune sera. (0.11 MB PDF) [file ppat.1000548.s002.pdf]
