# Supplementary material for: Two HIV-1 Variants Resistant to Small Molecule CCR5 Inhibitors Differ in How They Use CCR5 for Entry
Source: PLoS Pathog. 2009 Aug 14;5(8):e1000548. doi: 10.1371/journal.ppat.1000548 (PMC2718843; doi:10.1371/journal.ppat.1000548)
Supplement: Table S1 — Summary of some phenotypic characteristics of CCR5 inhibitor-sensitive and -resistant viral clones. (0.07 MB PDF) [file ppat.1000548.s003.pdf]

**Table S1.** Summary of some phenotypic characteristics of CCR5 inhibitor-sensitive and -resistant viral clones

| **Clone name** | **Site of resistance mutations** | **CCR5 inhibitor sensitivity** | **sCD4 sensitivity** | **Dependence on CCR5 NT** | **CD4i epitope exposure** | **V3 epitope exposure** |
|---|---|---|---|---|---|---|
| CC1/85 cl.7 | N/A | + | + | - | - | -* |
| CC1/85 cl.6 | N/A | + | - | - | - | - |
| CC101.19 cl.7 | gp120 V3 | - | + | + | + | + |
| D1/85.16 cl.23 | gp41 FP | - | - | - | - | - |

* This clone was partially sensitive to neutralization with V3 MAbs.

Note that the terminology used (+ *vs* -) is non-quantitative and does not denote absolutes; a listing of (+) means that the phenotype is more pronounced than when (-) is recorded.
