# Supplementary material for: Two HIV-1 Variants Resistant to Small Molecule CCR5 Inhibitors Differ in How They Use CCR5 for Entry
Source: PLoS Pathog. 2009 Aug 14;5(8):e1000548. doi: 10.1371/journal.ppat.1000548 (PMC2718843; doi:10.1371/journal.ppat.1000548)
Supplement: Text S1 — Supporting results and methods. (0.09 MB PDF) [file ppat.1000548.s004.pdf]

Supporting Results

Generation of rabbit anti-V3 peptide antisera

To create additional antibody probes for studying the antigenic structure of CC101.19 cl.7, we immunized rabbits (two per group) with 34-residue V3 peptides derived from this virus and also from CC1/85 cl.7, which has the same V3 sequence as CC1/85 cl.6 and D1/85.16 cl.23. The antisera obtained 49 days after the initial immunization were reactive with both the cognate and non-cognate peptides in an ELISA, although the titers against the non-cognate peptide were generally lower by ~0.5 log (Fig.S1). Binding titers were also determined against monomeric gp120 proteins derived from CC1/85 cl.7 and CC101.19 cl.7, using a gp120-capture ELISA. The two sera raised against the V3-CC1/85 peptide were unable to bind CC101.19 cl.7 gp120, presumably as a result of its 4 sequence changes in V3 (Fig.S2B). However, serum R1 also bound poorly to the cognate gp120, consistent with its low reactivity with the V3 peptides (Fig.S2A, compare with Fig.S1). Conversely, antisera to the V3-CC101.19 peptide bound to both gp120s, although more strongly to the cognate gp120 (Fig.S2B). Both V3 peptides are, therefore, immunogenic in rabbits, inducing antibodies that are gp120-reactive.

Phenotypic properties of parental and CCR5 inhibitor-resistant viruses

Some properties of the clones used in this study are summarized in Table S1.

Supporting Methods

Immunization of rabbits with V3 peptides

The 34-residue V3 peptides were derived from the sequences of CC1/85 or CC101.19. V3-CC1/85 and V3-CC101.19 peptides have the same sequences as the ones represented in Fig.1C, but lack the N-terminal cysteine residue. The peptides were synthesized, HPLC-purified and conjugated to KLH by Pocono Rabbit Farm and Laboratory (Canadensis, PA).

Specific pathogen free (SPF) New Zealand White female rabbits were immunized intradermally with 200 μg of the KLH-conjugated peptides on day 0, using complete Freund's adjuvant and a proprietary Immune Stimulator according to the Quick Draw Rabbit Polyclonal Antibody Protocol (Pocono Rabbit Farm and Laboratory). Rabbits R1 and R2 received the V3-CC1/85 peptide, R3 and R4 V3-CC101.19. Booster immunizations with 100 μg of the KLH-conjugated peptides in incomplete Freund's adjuvant were administered subcutaneously on days 7, 14 and 35. Blood was collected prior to immunization and on days 21, 28, 35, 42, 49 and 52.

V3 peptide ELISA

In brief, 100 μl of each V3 peptide (1 μg/ml) were coated onto microplate wells (Immulux HB, Dynex, Chantilly, VA) overnight at 4°C in sodium carbonate buffer. The plates were washed with PBS/0.1% Tween, and then blocked with PBS/10% FBS for 2h. Serum samples were serially diluted in blocking buffer and added to the wells for 1h at room temperature. After washing with PBS/0.1% Tween, an HRP-conjugated anti-rabbit-IgG + IgM detection antibody (SouthernBiotech, AL) was added for 1h at RT. After washing with PBS/0.1% Tween, the colorimetric endpoint was determined at 490nm using the AMPAK system according to the manufacturer's recommendation (OXOID, Cambridgeshire, UK). For each serum dilution, the OD_{490} value derived using pre-immune serum was subtracted from the value derived using test serum.

Non-linear sigmoidal dose-response curves were generated with Prism software. End-point titers were calculated as follows: $X = \log EC_{50} - ([\log(TOP/Y-1)/Hillslope])$, where Y = 5 SD of 3 background wells receiving V3 peptides but no serum and X = log[serum dilution] (the $\log EC_{50}$, TOP and Hillslope parameters were calculated using Graphpad Prism software). Midpoint titers (EC_{50} values) were calculated using Prism.

gp120 capture ELISA

The procedure was identical to that outlined in the main text, except that rabbit immune or pre-immune sera diluted in TSM were used, and the HRP-labeled detection antibody was specific for rabbit antibodies.
